# Which experimental factors govern successful animal-to-human translation in multiple sclerosis drug development? A systematic review and meta-analysis

**DOI:** 10.1016/j.ebiom.2024.105434

**Published:** 2024-11-07

**Authors:** Ingrid Berg, Pia Härvelid, Wolfgang Emanuel Zürrer, Marianna Rosso, Daniel S. Reich, Benjamin Victor Ineichen

**Affiliations:** a Center for Reproducible Science, University of Zurich, Zurich, Switzerland; b Clinical Neuroscience Center, University of Zurich, Zurich, Switzerland; c National Institute of Neurological Disorders and Stroke, National Institutes of Health, Bethesda, MD, USA

**Keywords:** Multiple sclerosis, Experimental autoimmune encephalomyelitis, Systematic review, Meta-analysis, Drug development, Translation

## Abstract

**Background:**

Despite successes in multiple sclerosis (MS) drug development, the effectiveness of animal studies in predicting successful bench-to-bedside translation is uncertain. Our goal was to identify predictors of successful animal-to-human translation for MS by systematically comparing animal studies of approved disease-modifying therapies (DMTs) with those that failed in clinical trials due to efficacy or safety concerns.

**Methods:**

Systematic review of animal studies testing MS DMTs, identified from searches in PubMed and EMBASE. A random effect meta-analysis was fitted to the data to compare outcome effect sizes for approved versus failed DMTs. Effect sizes and testing under diverse experimental conditions were assessed as potential predictors for successful translation.

**Findings:**

We included 497 animal studies, covering 15 approved and 11 failed DMTs, tested in approximately 30′000 animals. DMTs were tested in a small repertoire of experimental parameters: about 86% of studies used experimental autoimmune encephalomyelitis (EAE), 80% used mice, and 76% used female animals. There was no association between animal study outcomes or testing DMTs under varied conditions (e.g., different laboratories or models) and successful approval. Surprisingly, 91% of animal studies were published after first-in-MS trial and 91% after official regulatory approval.

**Interpretation:**

Our findings emphasize the complexity in carrying drugs from animals to clinical practice. Specific challenges include limited experimental methods in animal research and a disconnect between preclinical and clinical research. We advocate for efforts to streamline drug development for MS to improve animal research's relevance for patients.

**Funding:**

10.13039/100000002NIH, 10.13039/100000001Swiss National Science Foundation, 10.13039/501100000733Universities Federation for Animal Welfare.

**Research in context**

**Evidence before this study**

It remains unclear how well findings from animal studies predict the successful clinical application of drugs for multiple sclerosis (MS). To address this, we conducted a systematic review including literature searches in PubMed and EMBASE up to June 08, 2024, using terms related to MS animal models and the development of disease-modifying therapies (DMTs). Our search covered both DMTs that have been approved for MS treatment and those that failed in clinical trials due to efficacy or safety reasons. We included all original studies involving any eligible DMT in an MS animal model, excluding grey literature and review articles. We included 497 animal studies. Of these, 274 were analysed in a meta-analysis with the goal of identifying factors predicting successful translation from animal models to MS.

**Added value of this study**

Our systematic review and meta-analysis provide four main findings: (1) Preclinical MS drug research relies on a limited set of experimental methods, often using a narrow selection of animal models, species, and sex. (2) The diversity of experimental settings, such as conducting evaluations across multiple independent laboratories or utilizing various MS animal models does not seem to enhance the successful clinical application of DMTs. (3) Outcomes with a presumably high clinical relevance, including EAE scores or neuroimaging measures, are not associated with the successful clinical translation of MS treatments. (4) A substantial share of animal studies is published post-initial human trials or even after regulatory approval.

**Implications of all the available evidence**

Our findings underscore the complexity in translating drug efficacy from animal models to human application. To improve the impact of animal studies on patient care, we advocate for efforts to streamline preclinical multiple sclerosis drug development. Such strategies could include a stronger collaboration between preclinical and clinical researchers. This could ensure that animal research more accurately predicts and supports the development of effective treatments for MS.

## Introduction

In contrast to many other neurological diseases, the drug development landscape for multiple sclerosis (MS) has witnessed notable successes, with Food and Drug Administration (FDA)-approved drugs like glatiramer acetate owing their origin, at least in part, to foundational animal studies.^1^ However, in recent years, the path from bench to bedside has been more challenging: although some candidate drugs have shown encouraging results in preclinical studies, their translation to effective human treatments has been inconsistent.^2^

Two cases that underscore the challenges in translating preclinical promise to clinical application are those of natalizumab and opicinumab. Natalizumab, a humanised anti-α4 integrin antibody, initially demonstrated efficacy in preclinical experimental autoimmune encephalomyelitis (EAE) studies and subsequent phase 2 and 3 clinical trials in persons with MS.^3^ However, its post-approval clinical application faced a major setback when certain patients developed progressive multifocal leukoencephalopathy (PML), an opportunistic disease caused by the JC polyomavirus. This virus is prevalent in humans but is not found in rodents, underscoring a limitation in preclinical animal studies where such human-specific complications remain unanticipated.^4^^,^^5^ On a similar note, opicinumab, also known as the Lingo-1 antibody, had generated optimism based on its animal studies. The molecule was considered a potential therapeutic candidate for enhancing myelin and neuronal repair.^6^^,^^7^ Yet, in human trials, it failed to meet its primary endpoint, thus raising questions about the direct translation of its animal model efficacy to humans.^8^

Although advances in drug development for MS have led to the creation of several disease-modifying therapies (DMTs),^9^ the predictability of animal studies in anticipating successful clinical outcomes remains unclear. Specifically, the ability of preclinical metrics, such as effect sizes in animal models, to predict the subsequent efficacy and safety of DMTs in human trials is not yet well-understood. This uncertainty not only hinders efficient drug discovery but also raises ethical concerns about the number of animals employed in potentially noninformative research.^10^^,^^11^ MS is a particularly suited paradigm for this assessment as it presents both approved and failed drugs, allowing for a direct retrospective comparison of their respective animal studies.^12^, ^13^, ^14^ Thus, our study had the objective to identify potential factors within animal studies that could predict successful animal-to-human translation for MS. To achieve this, we systematically compared animal studies testing currently approved DMTs for MS against those DMTs that failed in MS clinical trials due to efficacy or safety reasons.

## Methods

### Protocol registration

We pre-registered the study protocol in the Open Science Framework (OSF, https://osf.io/sjnym/) and used the Preferred Reporting Items for Systematic Reviews and Meta-Analysis (PRISMA) 2020 Guidelines for reporting.^15^

### Search strategy and eligibility criteria

#### Identification of approved DMTs and DMTs that eventually failed in MS clinical trials due to safety or efficacy concerns

Approved DMTs were defined as listed on the National Multiple Sclerosis Society homepage (https://www.nationalmssociety.org/Treating-MS/Medications). Failed drug candidates were identified via two sources: 1) Adis Insight, a comprehensive drug development database (https://adisinsight.springer.com/), reporting exact timelines of drug development including reasons for failure; and 2) three reviews discussing failed and interrupted clinical trials of immunomodulatory treatment strategies for multiple sclerosis.^12^, ^13^, ^14^ The search was conducted in April 2023.

#### Inclusion criteria

Any DMT that is approved for MS care or that failed in any phase 0–4 MS clinical trial due to safety and/or efficacy reason. We counted Rituximab as approved even though it is used off-label in MS.^16^

#### Exclusion criteria

DMTs that were only tested in persons with progressive multiple sclerosis, i.e., imilecleucel-T and dirucotide (We excluded these since it has been observed that clinical trials evaluating DMTs for progressive MS may fail due to improper planning, rather than an inherent inefficacy of the DMTs themselves). Non-DMT drugs, i.e., to primarily treat MS symptoms.

#### Identification of animal studies testing approved/failed DMTs

We searched for animal studies up to June 08, 2024, in PubMed and EMBASE. Detailed search queries are provided in the Supplementary Data.

#### Inclusion criteria

Any original study that investigated any of the eligible DMTs in an MS animal model, i.e., anti-galactocerebroside antibodies, cuprizone, EAE, ethidium bromide, lysolecithin, or Theiler ׳s murine encephalomyelitis virus (TMEV). We included all languages.

#### Exclusion criteria

Studies that assessed a DMT *in vitro* only or in invertebrate animal species, grey literature (e.g., conference abstracts, book chapters); (systematic) reviews were excluded but retained as sources for additional references.

### Study selection, data extraction, and interrater agreement

Titles and abstracts were screened for relevance in the web-based application ASReview^17^ by one reviewer (IB), followed by a random sample check of 10% by the principal investigator BVI. ASReview incorporates an active-learning framework to present the most likely relevant record based on prior inclusion and exclusion decisions and thus making the screening process more efficient. We defined heuristic stopping criteria based on published recommendations for abstract screening augmented by machine learning^18^: The criteria were: 1) all predefined benchmark papers were marked as relevant (we had a set of 15 benchmark studies known to be relevant), 2) at least twice the number of relevant records identified in a pilot screening of a random subset (at least 1% of the full library) were screened (we found 8 relevant records in a pilot round of 100 random references), 3) a minimum of 10% of the total dataset was screened (from a total reference library of 7304 references), and 4) no relevant records were identified in the last 20 consecutive records. Screening was stopped once all these criteria were met. To assess the sensitivity of this approach, a reviewer screened the remaining unseen abstracts. No further eligible studies were identified, indicating a sensitivity of 100% for our screening method.

The following data were extracted:

#### Study characteristics

Study country, animal model, species, strain, sex, and age, the tested DMT, the overall study outcome (based on the authors conclusion, vote counting, i.e., negative, neutral, positive, mixed), total number of animals used in the study and per treatment group, treatment regimen (therapeutic or prophylactic), and potentially reported adverse events in animals upon DMT application.

#### Primary outcomes

EAE scores and MRI outcomes for treatment and control groups (including type of presented data, i.e., mean/median and variance measures). We selected these outcomes because of their presumable higher clinical relevance, as they resemble actual outcomes assessed in persons with MS and clinical trials.^19^ EAE scores resemble clinical disability, as measured by tools like the Expanded Disability Status Scale (EDSS), and MRI outcomes (i.e., measures of brain atrophy, lesions, contrast-enhancement, magnetization transfer imaging, and T1/T2) are commonly assessed in persons with MS, including in clinical trials. This contrasts with outcomes with less direct relevance for patients, such as histopathological assessments.

Data were extracted by three independent reviewers (IB, PH, WEZ). Interrater agreement was assessed on a random sample of 53 studies extracted in triplicate by the same three reviewers. Disagreement was resolved by discussion. Non-English articles were prioritised for translation by native speakers within our group; if this was not possible, Google Translate was used as a secondary option.

### Critical appraisal of included studies

Risk of bias was assessed using two approaches: first, against a pre-defined 6-item checklist: i.) Reporting of randomization ii.) Reporting of blinding, iii.) Reporting of an animal welfare statement, iv.) Statement of a potential conflict of interest, v.) Sample size calculations provided, and vi.) In accordance with the ARRIVE guidelines.^20^, ^21^, ^22^, ^23^ The risk of bias was automatically assessed by a validated text mining function in the R programming environment.^24^^[preprint]^ To assess performance of the automated tool, the risk of bias was manually assessed in 53 studies by three independent reviewers (IB, PH, WEZ; the same studies as for data extraction validation), and agreement was evaluated via F1-score, precision (positive predictive value), and recall (sensitivity). Second, in a random sample of 10% of eligible studies, we assessed the risk of bias using the SYRCLE risk of bias assessment tool.^25^

### Analysis of study reasoning

For all studies testing regulatory-approved DMTs, we analysed the authors' reasoning for conducting their respective studies. Based on the review of 30 studies, we pre-defined 10 criteria for the potential motivations behind the studies:

1. Mechanism: to better understand the DMT mechanism, e.g., how fingolimod affects T cells.
2. New DMT: using an approved DMT as an active control to benchmark against a new DMT.
3. DMT comparison: comparing two already approved DMTs against each other.
4. Animal model investigation: using a DMT to better understand a model disease in animals, e.g., testing a newly developed model to see if it benefits from established DMTs.
5. Regimen: Testing a new treatment regimen, e.g., different doses or prophylactic treatment.
6. Proof-of-principle efficacy: Establishing the initial efficacy of a DMT under investigation.
7. Translation: testing a DMT in animal models to explore its potential translation to humans.
8. Progressive MS: assessing the effect of a DMT on a progressive MS-animal model.
9. Toxicology: assessing the potential toxic effects of a DMT.
10. Reproducibility: testing whether prior findings using a DMT can be reproduced.

Each study was classified into one or more of these categories.

### Statistics

We conducted all statistical analyses in the R programming environment (version 4.2.2). Findings were summarised in a narrative fashion bolstered by descriptive statistics of extracted parameters. One meta-analysis was conducted per DMT and only for DMTs for which at least three studies reported either EAE scores or MRI outcomes. As primary outcome, Hedges' g standardised mean difference (SMD) was used which was pooled to obtain an overall SMD and 95% confidence intervals, using the R package *metafor* for the meta-analysis.^26^ A random-effects model was fitted to the data.^27^ The amount of heterogeneity, i.e., τ^2^, was estimated using the DerSimonian-Laird estimator. The Q-test for heterogeneity and the I^2^ statistic were calculated. An umbrella meta-analysis was calculated pooling SMDs for approved versus failed DMTs. Effect sizes between approved and failed DMTs were compared using the Wilcoxon test. We conducted sensitivity analyses only including studies reporting blinding and/or randomization, i.e., being at lower risk of bias.

To assess experimental factors from animal studies potentially associated with successful clinical translation, we *a priori* defined animal study characteristics that we thought could be associated with clinical translation, inspired by the Stroke Therapy Academic Industry Roundtable (STAIR) criteria.^28^ The criteria that we considered to be associated with successful translation were: whether a DMT was tested in two or more MS animal models, in both sexes, two or more species/strains, with at least two outcomes (EAE scores and MRI), in at least two independent laboratories, and/or with a therapeutic regimen (compared to prophylactic treatment). We qualitatively compared the proportions of these criteria between approved and failed DMTs without conducting statistical comparisons.

To assess development times from first-in-animal studies to first-in-MS studies, FDA approval, or failure in a clinical MS trial, we conducted a Kaplan–Meier survival analysis. For this, we used the R packages s*urvminer* (RRID: SCR_021094) and *survival* (RRID: SCR_021137).

To search for the evidence of publication bias, we used Funnel plots, Egger regression, and trim-and-fill analyses. Because SMDs may cause funnel plot distortion, we plotted the SMD against a sample size-based precision estimate (1/√(n)).^29^

A two-tailed p value < 0.05 was considered statistically significant. We corrected for multiple comparisons controlling the false discovery rate.^30^

### Deviations from the protocol

We initially included only English studies but later decided to extend the scope to include studies without language restrictions. We originally assessed the risk of bias using the CAMARADES checklist, and subsequently, we additionally employed the SYRCLE risk of bias assessment tool.^25^

### Ethics

This is a systematic review and meta-analysis and thus no study approval was required.

### Role of funders

The funders had no role in the design and conduct of the study; collection, management, analysis, and interpretation of the data; preparation, review, or approval of the manuscript; and decision to submit the manuscript for publication.

## Results

### Eligible DMTs and animal studies

Our search found 17 approved and 25 failed DMTs for relapsing-remitting MS (Table 1). Out of 6312 screened publications, 775 studies were eligible for full-text search. Finally, 497 studies were included for qualitative synthesis and 274 studies for the meta-analysis (Supplementary Figure S1). Animal studies covered 15 approved and 11 failed DMTs, with some studies evaluating multiple DMTs (Table 1).

**Table 1:** Disease modifying therapies (DMTs) that were approved (n = 17) or failed (n = 25) in MS clinical trials.

Approved DMTs (n = 17)	Failed DMTs (n = 25)
**Alemtuzumab** **Cladribine** **Dimethyl fumarate** **Fampridine** **Fingolimod** **Glatiramer acetate** **Interferon Beta 1** **Monomethyl fumarate** **Natalizumab** Ocrelizumab Ofatumumab **Ozanimod** **Peginterferon beta-1a** **Ponesimod** **Rituximab** **Siponimod** **Teriflunomide**	**Abatacept** Acyclovir **Atacicept** **Atorvastatin** Autologous T cell vaccine BGC-200134 BHT-3009 BX-471 Dirucotide Efalizumab **Epigallocatechin gallate** **Estriol** **Inosine** **Minocycline** **Opicinumab** Plozalizumab Rosiglitazone Rovelizumab Tabalumab Temelimab Tiplimotide **Toralizumab** **Ustekinumab** Vatelizumab **Vitamin D3**

DMTs for which we identified animal studies in multiple sclerosis (MS) animal models are in bold font (n = 15 approved DMTs, n = 11 failed DMTs), in alphabetical order.

### General study characteristics

#### Included publications and DMTs

**Fig. 1:**
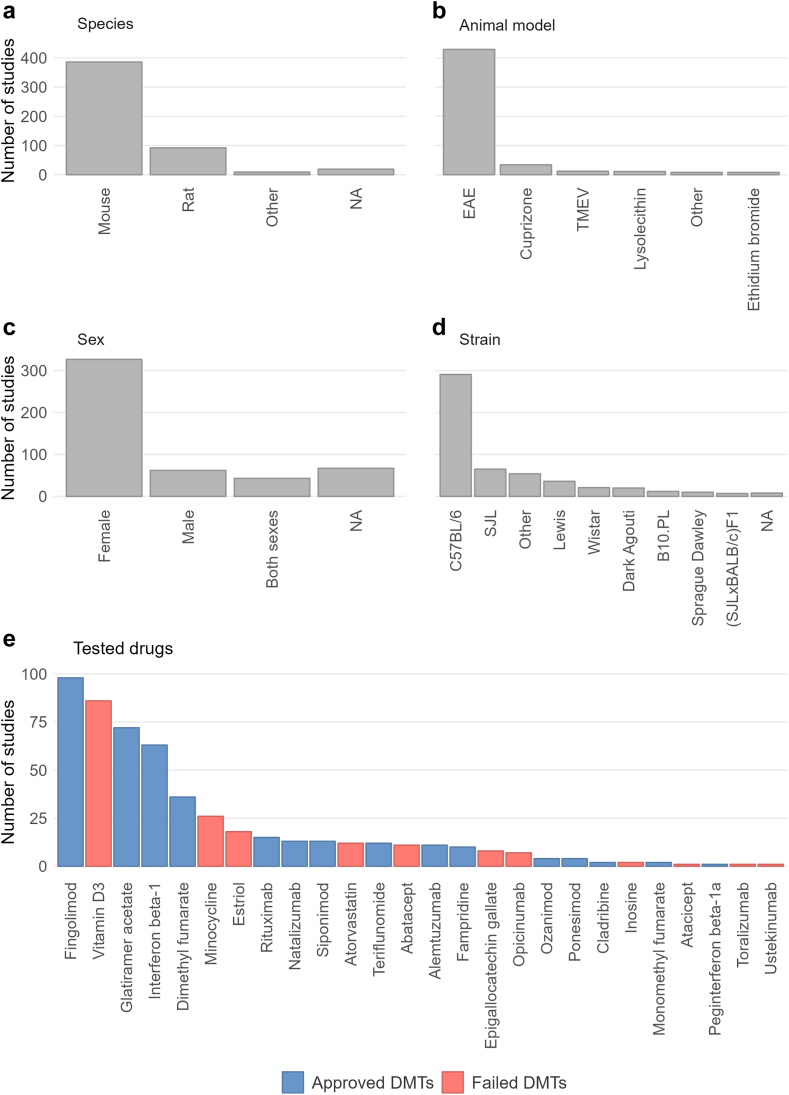
Animal study demographics and included disease-modifying therapies (DMTs). Number of studies per study characteristic: Used species (a), animal models (b), sexes (c), and strains (d) in multiple sclerosis (MS) drug development. Number of studies per included DMT (e), blue bars represent approved DMTs, and red bars failed DMTs. Abbreviations: EAE, experimental autoimmune encephalomyelitis; NA, not reported; TMEV, Theiler's murine encephalomyelitis virus.

For DMT testing, the most commonly used species were mice (80%, most common strain: C57BL/6, 72% of all mouse studies) and rats (19%, most common strain: Lewis, 40% of all rat studies) (Fig. 1). Female animals were more commonly used than males (324 studies, 76% of studies reporting sex). The most frequently employed MS animal model was EAE (425 studies, 86%), most commonly induced using myelin oligodendrocyte glycoprotein (MOG, 67%), followed by myelin basic protein (MBP) and proteolipid protein (PLP, each at 11%). Cuprizone was the second most commonly used MS animal model (35 studies, 7%). Most studies reported EAE scores (364 studies, 74%) and 17 reported MRI outcomes (3%). Only two studies reported potential adverse drug events in animals (<1%). The median number of animals used per study was 62, resulting in approximately 30,000 animals being used for the testing of approved and failed DMTs.

The random 10% sample check of 203 references indicated that the ASReview-enhanced manual screening had a sensitivity of 100% to identify eligible studies, i.e., the process identified all the records found by the sample check. The specificity was slightly lower at 89%, indicating that 162 out of 183 irrelevant records were correctly excluded during the process. The inter-rater agreement for data extraction ranged from 83% to 93%, depending on the specific item.

#### Risk of bias assessment

For the CAMARADES checklist, automated risk of bias assessment reached high performance metrics for most items with F1-scores >80% (Supplementary Table S1). Potential conflict of interest showed low performance and was thus excluded from the automated risk of bias assessment. Many studies reported an animal welfare statement, with 76% complying with local guidelines. Yet only 35% and 34% of the animal studies reported blinding or randomization, respectively. Additionally, only 6% of studies reported an *a priori* sample size calculation and 4% reported following ARRIVE guidelines (Supplementary Figure S2a).

For the SYRCLE risk of bias assessment, 30% of the studies reported random outcome assessment, indicating a relatively low risk of detection bias. Similarly, 28% of the studies reported blinding of experiments, suggesting a moderate risk of performance bias. However, 46% of the studies had a high risk of bias due to funding from pharmaceutical industry, constituting a potential conflict of interest. The other bias domains had mostly unclear risk of bias (Supplementary Figure S2b).

The inter-rater agreement for risk of bias assessment ranged from 79% to 91%, depending on the specific item.

### Effect sizes do not differ between animal studies testing approved versus failed DMTs

**Fig. 2:**
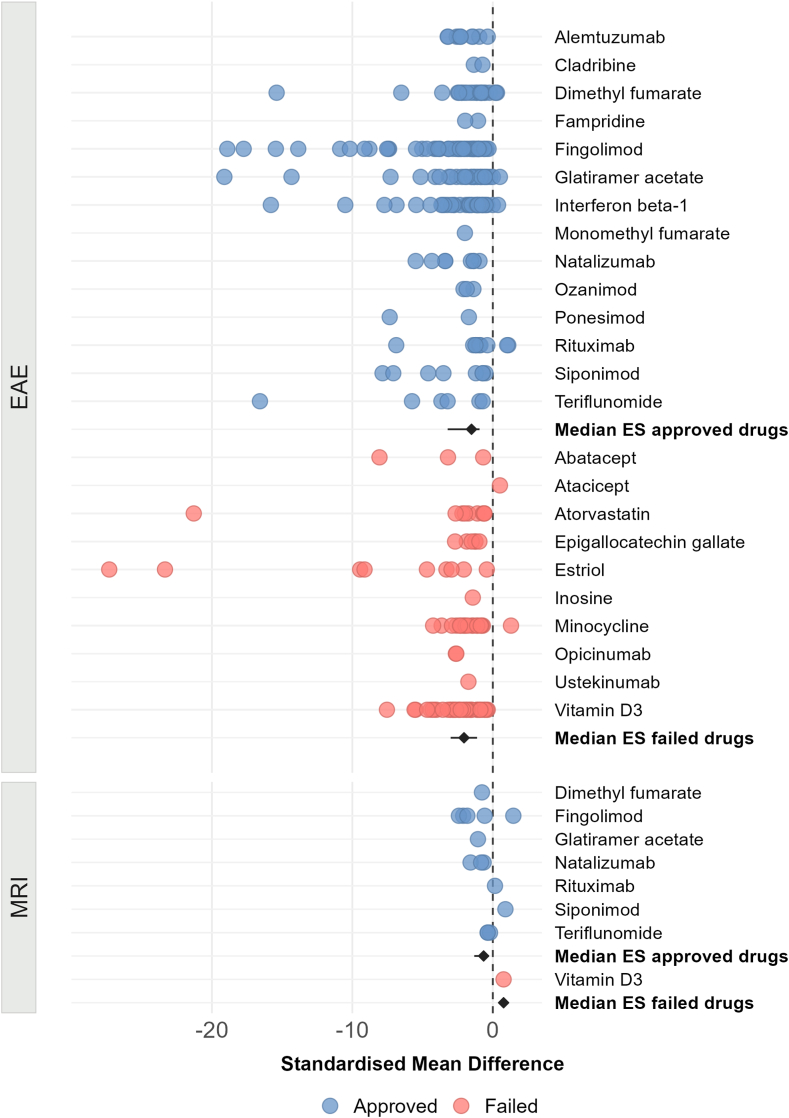
Effect sizes for EAE scores and MRI outcomes for approved and failed disease-modifying therapies (DMTs). Hedges' g standardised mean differences per DMT and study and pooled for both EAE scores (top panel) and MRI outcomes (bottom panel). Pooled effect sizes for approved and failed DMTs are similar. Error bars represent 95% confidence intervals. Abbreviations: EAE, experimental autoimmune encephalomyelitis; ES, effect sizes; MRI, magnetic resonance imaging.

SMDs were calculated from reported EAE scores and MRI outcomes. EAE score median effect sizes of approved and failed DMTs were −1.52 [interquartile range: −3.21 to −0.94] and −2.05 [range: −3.00 to −1.12], respectively. MRI outcome effect sizes of approved and failed DMTs were −0.66 [range: −1.32 to −0.28] and 0.76 (one DMT), respectively. There was no statistically significant difference between approved and failed DMT EAE score effect sizes (Wilcoxon: p = 0.14) (Fig. 2). The difference remained non-significant in a sensitivity analysis when only including animal studies with lower risk of bias (i.e., blinded and/or randomised; Wilcoxon: p = 0.24) (Supplementary Figure S3).

### Meta-analysis per DMT showed a beneficial effect for both approved and failed DMTs

**Fig. 3:**
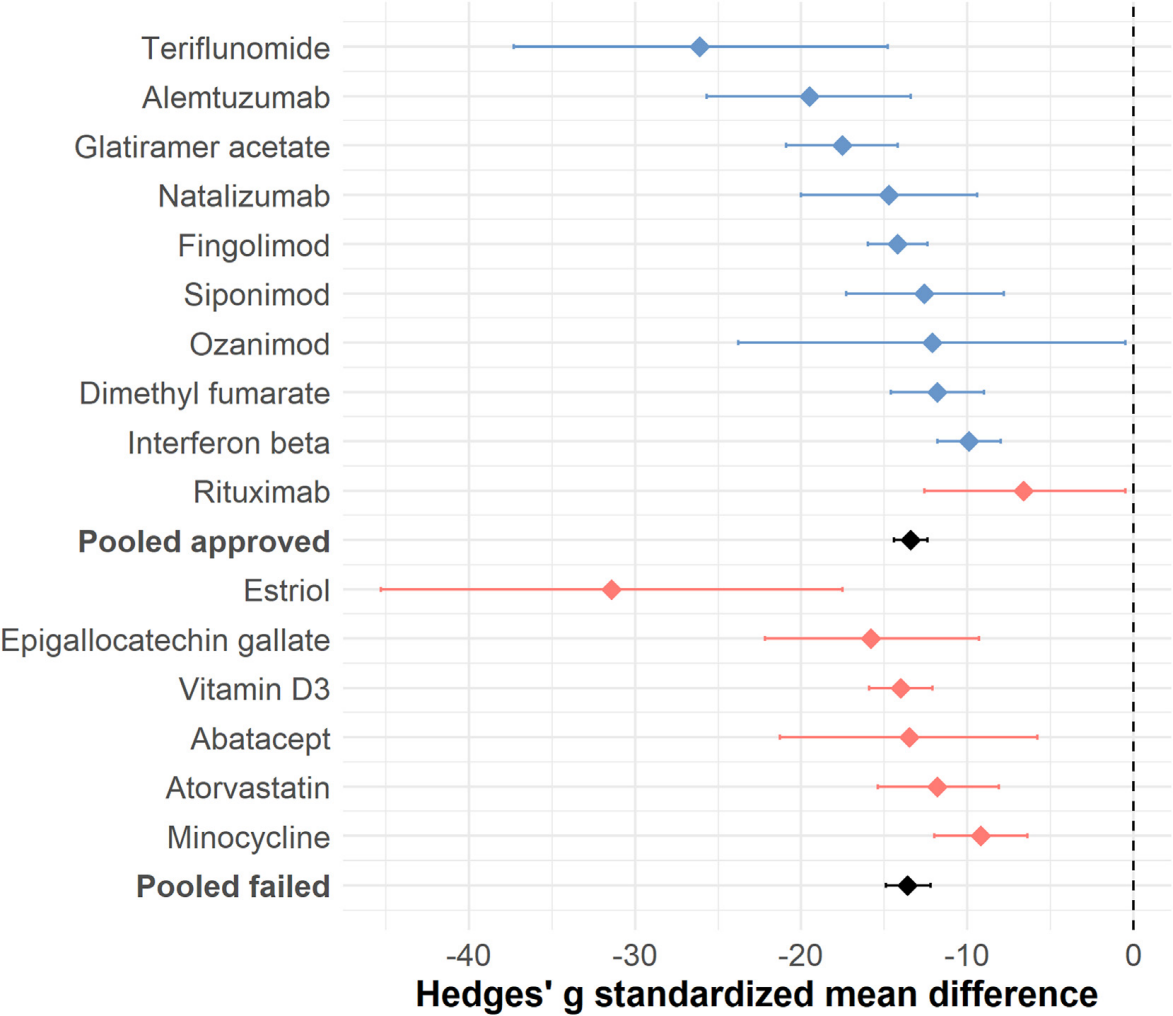
Umbrella forest plot to compare pooled effect sizes of EAE scores between approved and failed disease-modifying therapies (DMTs). Pooled analyses of studies showing the effect of DMTs (versus control) on EAE scores, stratified by DMT (blue = approved DMTs, red = failed DMTs), including overall pooled estimates (black). The diamonds indicate the global estimates. bars indicate the 95% confidence interval (CI) of the overall effect size. None of the bars overlaps with 0, i.e., both approved and failed DMTs showed a beneficial effect on EAE scores.

Sixteen DMTs were eligible for a meta-analysis (10 approved and 6 failed), i.e., presenting EAE scores or MRI outcomes in at least 3 studies (Table 2 and Supplementary Table S2). For EAE scores, all 16 pooled meta-analyses suggested a statistically significant beneficial effect of DMT application on clinical disability of affected animals (Supplementary meta-analyses). Pooled effect sizes ranged for approved DMTs from −26.1 (teriflunomide) to −6.6 (rituximab) and for failed DMTs from −31.4 (estriol) to −9.2 (minocycline) (Table 2). An umbrella meta-analysis pooling EAE scores of all approved versus all failed DMTs showed similar pooled effect sizes for approved and failed DMTs: −13.9 [95%-CI: −15 to −12.8] for approved DMTs versus −13.6 [95%-CI: −15.6 to −12.3] for failed DMTs (Fig. 3) (Wilcoxon: p = 0.84). The difference remained non-significant when only including animal studies with lower risk of bias (i.e., blinded and/or randomised): −12.6 [95%-CI: −13.8 to −11.4, I^2^ = 95%] for approved DMTs versus −15.7 [95%-CI: −18.1 to −13.4, I^2^ = 97%] for failed DMTs (Wilcoxon: p = 0.31). The meta-analyses showed substantial heterogeneity (I^2^ for approved DMTs: 96%, for failed DMTs: 95%, Table 2). Heterogeneity remained substantial when only including mice data in the meta-analyses (I^2^ for approved DMTs: 96%, for failed DMTs: 95%).

**Table 2:** Meta-analysis and publication bias of EAE scores of approved and failed DMTs.

Drug	Number of studies	Effect sizes [95%-CI] (random effects model)	Heterogeneity I^2^	Number of studies missing (trim-and-fill)
**Approved**				
Teriflunomide	5	−26.1 [−37.3 to −14.8]	97%	1
Alemtuzumab	10	−19.5 [−25.7 to −13.4]	96%	0
Glatiramer acetate	25	−17.5 [−20.9 to −14.2]	97%	3
Natalizumab	7	−14.7 [−20.0 to −9.4]	95%	2
Fingolimod	61	−14.2 [−16.0 to −12.4]	94%	17
Siponimod	7	−12.6 [−17.3 to −7.8]	91%	0
Ozanimod	3	−12.1 [−23.8 to −0.5]	96%	2
Dimethyl fumarate	24	−11.8 [−14.6 to −9.0]	96%	0
Interferon Beta	35	−9.9 [−11.8 to −8.0]	94%	8
Rituximab	9	−6.6 [−12.6 to −0.5]	97%	0
**Pooled**	**186**	**−13.4 [**−**14.4** to −**12.4]**	**96%**	**33**
**Failed**				
Estriol	8	−31.4 [−45.3 to −17.5]	95%	0
Epigallocatechin gallate	6	−15.8 [−22.2 to −9.3]	91%	0
Vitamin D3	35	−14.0 [−15.9 to −12.1]	97%	12
Abatacept	4	−13.5 [−21.3 to −5.8]	93%	1
Atorvastatin	9	−11.8 [−15.4 to −8.1]	88%	3
Minocycline	17	−9.2 [−12.0 to −6.4]	93%	5
**Pooled**	**79**	**−13.6 [**−**14.9** to −**12.2]**	**95%**	**21**

One meta-analysis was conducted per DMT and only for DMTs for which at least three studies reported experimental autoimmune encephalomyelitis (EAE) scores. Data presented is Hedges' g standardised mean difference (SMD) and the I^2^ as a measure of heterogeneity. Number of missing studies is presented as computed via trim-and-fill analyses.

For MRI outcomes, 2 approved DMTs were eligible (fingolimod and natalizumab). Similar to EAE scores, both meta-analyses suggested a statistically significant beneficial effect of DMT application on MRI outcomes (Supplementary meta-analyses and Supplementary Table S2). Pooled effect sizes were −10.5 [95%-CI: −18.5 to −2.4] for fingolimod and −3.2 [95%-CI: −6.1 to −0.2] for natalizumab. The meta-analyses showed substantial heterogeneity (I^2^ = 95%).

### Similar degrees of publication bias for both approved and failed DMTs

Both visual inspection of the funnel plots and Egger's regression test suggested the presence of publication bias (Table 2, Supplementary Table S2, and Supplementary funnel plots). Trim-and-fill analyses suggested a total of 54 missing studies (95%-CI: 46–58 missing studies; 33 for a total of 6 approved DMTs and 21 for a total of 4 failed DMTs).

### Testing DMTs under more diverse experimental conditions was not associated with approval

Twenty-six DMTs were eligible for modelling potential animal study parameters associated with DMT approval or failure for MS (15 approved DMTs tested in 362 studies and 11 failed DMTs tested in 178 studies). Overall, animal studies testing eventually approved DMTs consistently showed higher proportions of experimental factors potentially associated with successful DMT approval, i.e., approved DMTs were more commonly tested in two or more MS animal models (53% versus 36%), in two or more species (60% versus 55%) and strains (66% versus 64%), in both sexes (73% versus 55%), in studies with two or more outcomes (at least EAE scores and MRI, 47% versus 18%), and in at least two independent laboratories (100% versus 73%) (Table 3, top part). However, this did not hold true in a sensitivity analysis that considered only animal studies published before the initiation of the first clinical trial for the DMTs (Table 3, bottom part). A more detailed overview per DMT is presented in Supplementary Table S3.

**Table 3:** Experimental characteristics of animal studies testing approved and failed DMTs for MS.

	Approved DMTs	Failed DMTs
**Overall**		
Number of DMTs	15	11
Total number of studies	362	178
Mean number of animals per study	60	49
Number (%) of DMTs:		
Tested in two or more MS animal models	8 (53%)	4 (36%)
Tested in both sexes	11 (73%)	6 (55%)
Tested in two or more species	9 (60%)	6 (55%)
Tested in two or more strains	10 (66%)	7 (64%)
Tested with at least two outcomes (EAE score and MRI)	7 (47%)	2 (18%)
Tested in at least two independent laboratories	15 (100%)	8 (73%)
Tested with a therapeutic regimen (compared to prophylactic)	13 (87%)	9 (82%)
The mean proportion of animal studies with a beneficial outcome	0.89	0.83
**Before first-in-MS trial**		
Number of DMTs	6	8
Total number of studies	14	34
Mean number of animals per study	65	58
Number (%) of DMTs:		
Tested in two or more MS animal models	0 (0%)	1 (13%)
Tested in both sexes	1 (17%)	4 (50%)
Tested in two or more species	1 (17%)	3 (38%)
Tested in two or more strains	1 (17%)	6 (75%)
Tested with at least two outcomes (EAE score and MRI)	2 (33%)	1 (13%)
Tested in at least two independent laboratories	1 (17%)	7 (88%)
Tested with a therapeutic regimen (compared to prophylactic)	3 (50%)	7 (88%)
The mean proportion of animal studies with a beneficial outcome	1	0.92

Comparison between approved and failed disease-modifying therapies (DMTs) regarding their animal testing. Top part of table: Overall, DMTs that were eventually approved were more frequently tested in two or more MS animal models, in both sexes, in two or more species/strains, with at least two outcomes (EAE scores and MRI), in at least two independent laboratories, and with a therapeutic regimen (compared to prophylactic treatment). On average, a higher proportion of animal studies reported beneficial outcomes for DMTs that obtained approval. Bottom part of table: When considering only animal studies conducted prior to the respective first-in-MS trial, DMTs that later failed in MS clinical trials showed a broader set of experimental parameters.

Criteria are adjusted from the Stroke Therapy Academic Industry Roundtable (STAIR) criteria.^28^

Abbreviations: DMT, disease modifying therapies; EAE, experimental autoimmune encephalomyelitis; MRI, magnetic resonance imaging; MS, multiple sclerosis.

### Lengthy development timelines for MS DMT development

**Fig. 4:**
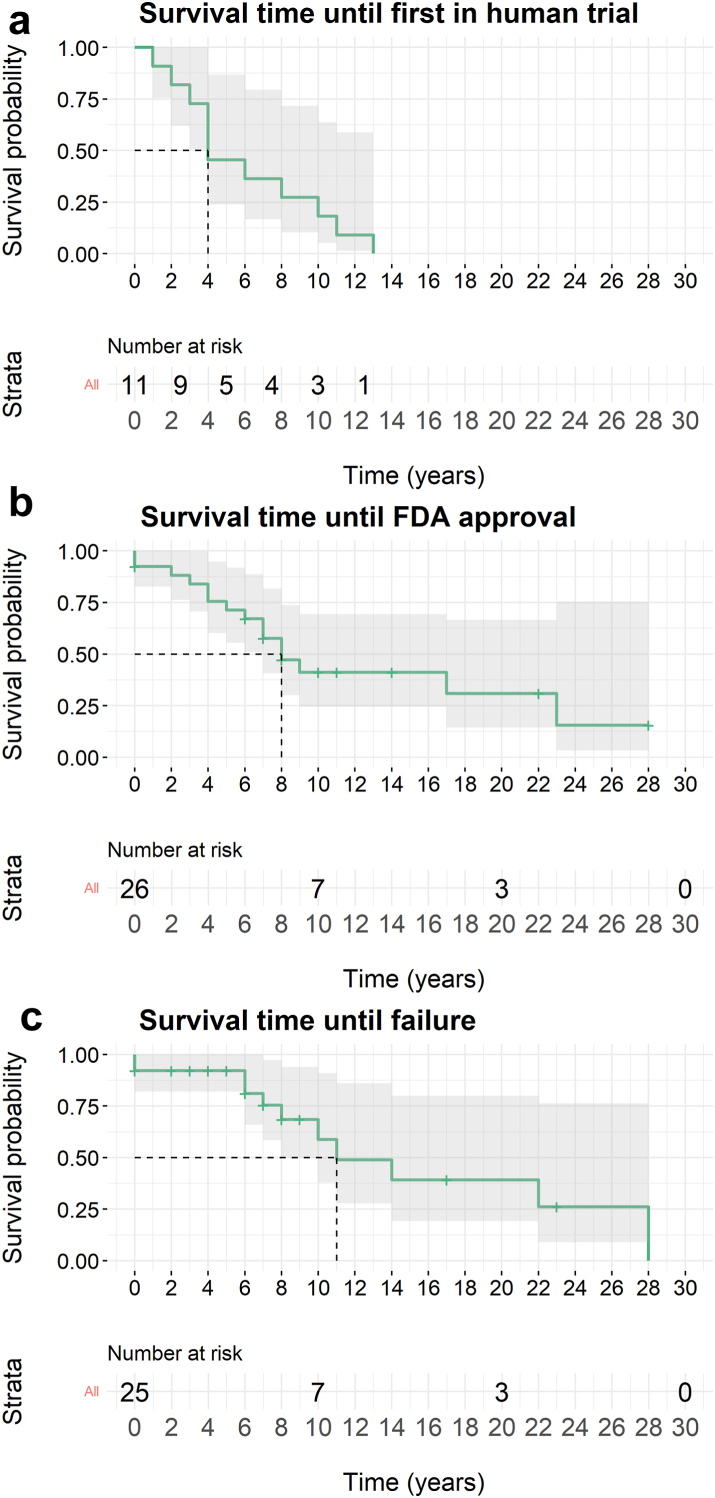
Kaplan–Meier survival analysis for DMT development. Kaplan–Meier survival curves depict the timelines for disease-modifying therapies (DMTs) achieving various milestones: first appearance in an MS trial (a, n = 11), FDA approval (b, n = 22), and clinical development failure (c, n = 19). For both FDA approval and clinical development failure, DMTs are censored at the respective time points when they either failed or achieved FDA approval. Median survival times are indicated by the dotted line. The strata present DMTs at risk per time point. Abbreviations: FDA, Food and Drug Administration.

The median first animal trial was in 2005 (range: 1979–2019). The median first human MS trial was in 2006 (range: 1977–2019). Years for approved and failed drugs are presented in Supplementary Table S4. The median time from first-in-animal study to first-in-MS trial, FDA approval, or clinical failure was 4 years (95%-CI: 4–not estimable), 9 years (95%-CI: 7–non estimable), and 11 years, respectively (95%-CI: 10–non estimable) (Fig. 4).

### Large majority of animal studies published after clinical trials and regulatory approval

**Fig. 5:**
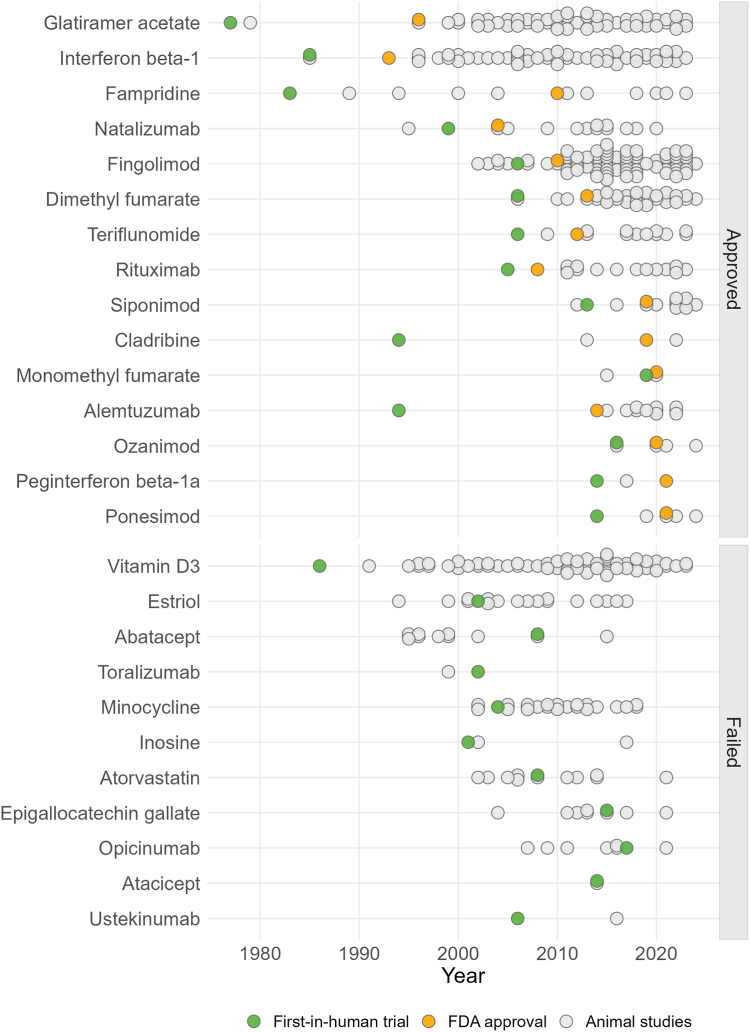
Timeline of disease-modifying therapies (DMTs) for multiple sclerosis. Approved DMTs are presented in the top panel, failed DMTs in the bottom panel. Grey circles represent individual animal studies, green circles represent first-in-MS trials and orange circles represent FDA-approval. Of note, rituximab has not obtained official FDA-approval.^31^ Abbreviations: FDA, Food and Drug Administration.

Published animal studies were predominantly undertaken after the initiation of first-in-MS trials. There were even instances, such as with glatiramer acetate and alemtuzumab, where no preceding animal studies were identified prior to these trials. Specifically, 91% of the animal studies were published after the first-in-human trials (450 of 497 studies), and 91% (300 of 329 studies) even after regulatory approval (Fig. 5). Collectively, these studies involved approximately 27′000 animals.

**Fig. 6:**
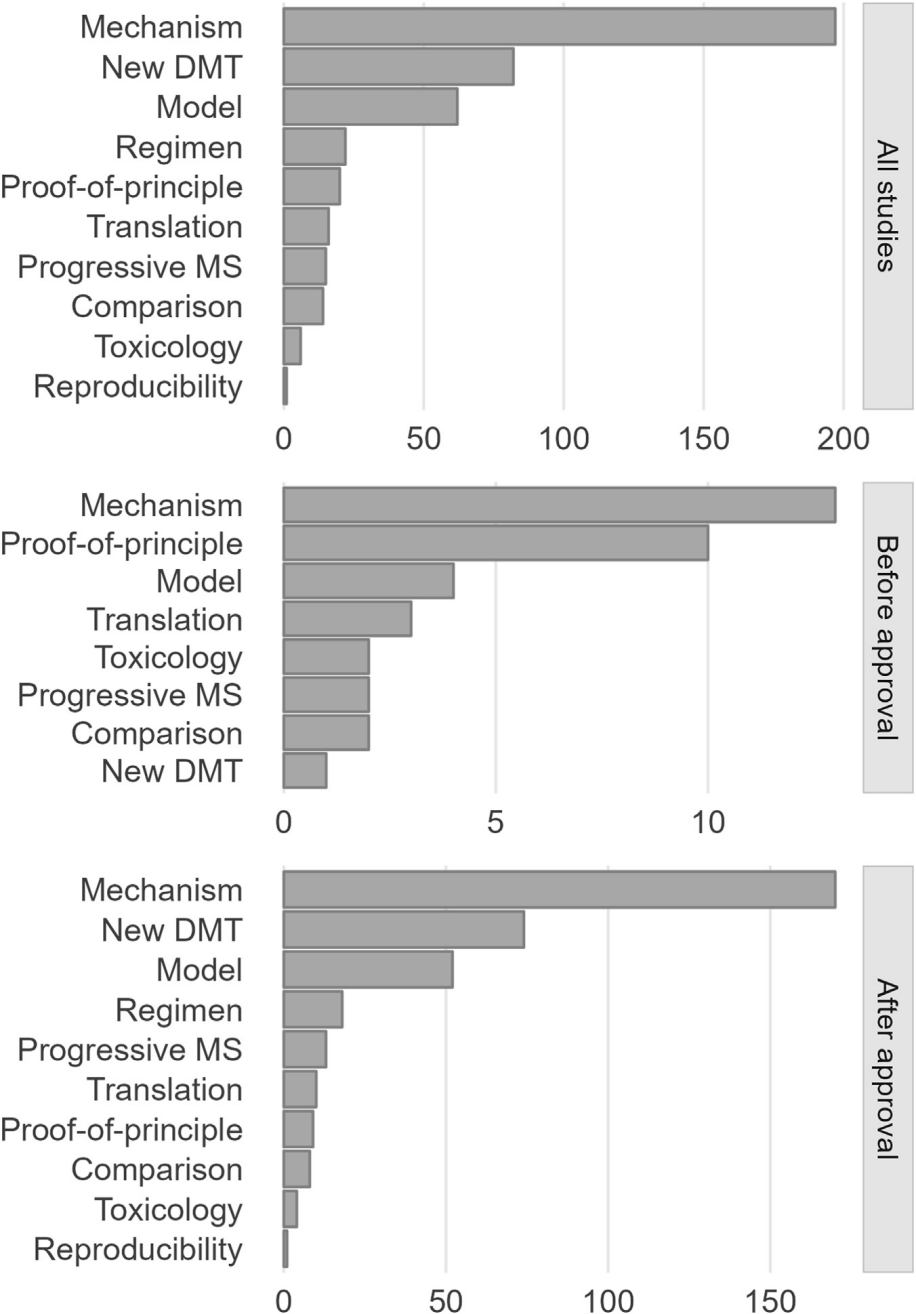
The authors' reasoning for conducting animal studies on regulatory-approved disease-modifying therapies (DMTs). Study authors rationale for conducting their study overall (top panel), prior to regulatory approval (middle panel), and after regulatory approval (bottom panel). A detailed description of each class is provided in the methods section. Abbreviations: Disease-modifying therapies (DMTs); MS, multiple sclerosis.

The authors' reasoning for conducting studies on regulatory-approved DMTs showed that the three most common motivations were: understanding a DMT's mode of action (197 studies, 60%), benchmarking a novel DMT candidate against an approved DMT (82, 25%), and using a DMT to establish a presumed MS animal model (62, 19%) (Fig. 6). For studies published before regulatory approval, the top reasons were almost similar (13 studies for better mechanistic understanding [52%], 4 for establishing a new model [16%], but also 10 for proof-of-principle efficacy [40%]; Fig. 6). After regulatory approval, the most common reasons remained mechanistic understanding, benchmarking novel DMTs, and establishing new MS animal models (Fig. 6). Notably, in post approval studies, 10 [4%] and 9 studies [3%] focused on translating a potential DMT or establishing proof-of-principle efficacy despite the DMT already being approved. The USA, Germany, and Israel were the countries conducting the most post-approval animal studies (Supplementary Figure S4).

## Discussion

### Main findings

Our systematic review and meta-analysis had the objective to assess the differences between animal experiments for MS-approved DMTs versus DMTs that failed in MS clinical trials. With this, we aimed to identify factors in animal research that might predict successful translation to human applications for MS treatments. Our data indicate that:

1. In preclinical MS drug research, there is a highly limited repertoire of experimental methods. For instance, a small consistent set of animal models and species is continually selected for evaluating putative DMT candidates.
2. Testing a DMT in diverse experimental settings was not associated with regulatory approval for MS.
3. Even seemingly clinically relevant outcomes, such as EAE scores or neuroimaging metrics that resemble those used in MS clinical trials, fail to predict successful translation to MS treatment.
4. A large majority of animal studies are published after initial human trials or even following official regulatory approval.

### Findings in the context of existing evidence

Preclinical MS DMT development predominantly uses a narrow set of experimental methods, with the majority of studies focusing on the EAE model, mice as the primary species, and predominantly using female animals. This approach has several limitations: First, while the EAE model is often used for MS research, its ability to fully represent MS's complex pathology, including inflammation, demyelination, and neurodegeneration, is debated.^32^^,^^33^ EAE primarily reflects neuroinflammation but falls short in accurately mimicking demyelination and myelin repair. Other models like lysolecithin or cuprizone may better mimic MS's demyelinating aspects, especially for assessing putative myelin regenerating treatments.^21^ The EAE model also fails to represent the progressive MS phenotype,^34^ often resembling conditions like acute disseminated encephalomyelitis (ADEM) instead.^32^ Second, although mice dominate these studies (like in most other branches of drug development^35^), in part likely due to their genetic similarity to humans and the ability to manipulate their genomes,^36^ this heavy reliance introduces limitations. For instance, mice have distinct physiological and immunological differences.^35^ Third, the predominance of female animals is likely linked to the fact that EAE can be induced more easily in female animals compared to male animals.^37^ This female bias in preclinical MS research stands in contrast with the common male bias in many biological research fields, including neuroscience.^38^ Importantly, sex can have a substantial impact on the MS disease course, partly driven by hormonal differences,^39^ and, thus, DMTs should in most cases be tested in both sexes. Together, multiple factors contribute to this narrow methodological repertoire. And this also includes “scientific inertia” — the adherence to familiar, traditional scientific practices.^40^^,^^41^ Furthermore, some experimental choices made for simplicity may not be ideal for clinical translation, such as the preference for mice due to their small size and ease of maintenance in animal facilites.^42^

Our study indicates that using a broader set of experimental approaches, such as different animal models, sexes, or testing in independent laboratories, does not necessarily benefit the successful development of DMTs for MS. Several initiatives aiming at improving animal-to-human translation have been launched, including the STAIR criteria in stroke research,^28^^,^^43^ which offer guidelines for preclinical drug testing, such as validating a potential drug's efficacy in multiple laboratories or in more than one species. Similar efforts to enhance the reliability and relevance of findings for human health have been recommended for conditions like epilepsy,^44^ myocardial infarction,^45^ and traumatic brain injury.^46^ Thus, despite our findings, we believe such endeavours are highly relevant, particularly given the narrow experimental portfolio used in preclinical MS drug development, as discussed above. This concept aligns with the so-called “standardization fallacy,” which posits that while standardization can enhance the power of research outcomes, it might also lead to unrealistically low variability.^47^ Consequently, findings from such a narrow set of experimental animal studies might not be applicable to human applications.

We show that apparently clinically relevant animal outcome measures such as EAE scores (mimicking clinical disability) or MRI outcomes may not be the best predictors of how a treatment will perform in human patients as both approved and failed showed similarly positive outcomes in our analyses. Of note, both approved and failed DMTs showed a similar degree of publication bias. Hence, publication bias is likely not responsible for an overestimated positive effect for failed DMTs. This underscores the importance of carefully selecting outcome measures.

Our findings support the idea that drug development often lacks a clear translational road map, even for successfully translated treatments.^10^ We found a large majority of animal studies published after initial human trials or following regulatory approval. Remarkably, for some drugs like alemtuzumab, cladribine, and ustekinumab, no preceding animal studies were found. However, not all clinical trials necessitate prior animal research, as seen with rituximab, initially developed for cancer and rheumatoid arthritis and later used off-label for MS.^31^ Nevertheless, 9 out of 10 animal experiments were published after regulatory approval, involving over 27,000 animals. There may of course be valid reasons for conducting animal experiments post initial human trials or even post-approval, such as seeking deeper understanding of a drug's mechanisms of action—a commonly observed rationale in our analysis. Such enhanced mechanistic understanding could potentially be used to develop new, more effective drugs. Nonetheless, some of the included animal studies aimed at translating already approved therapies for MS. This indicates that animal research may not always be driven by clinical needs but by separate research agendas. This gap between preclinical and clinical research can be attributed to various factors, including the arduous and risky nature of developing clinical applications, combined with the substantial resources and time required for translation.^48^^,^^49^ It also seems that animal researchers might not always be fully informed about ongoing clinical activities or even a drug's official approval status. This warrants an attempt at not only reconnecting these two fields, e.g., through the creation of dedicated funding mechanisms, but also to foster more evidence-based preclinical research to minimise research waste.^11^

### Limitations

Our study has notable limitations: First and foremost, 91% of animal studies we analysed were published after the DMT had received regulatory approval. This timing raises concerns about our ability to clearly differentiate between the cause and effect of the predictors we assessed. We mitigated this by a sensitivity analysis that included only animal studies carried out before regulatory approval. Additionally, since it is challenging to determine when an animal study was exactly conducted, we used the publication year as a proxy. This approach might lead to an overestimation of the proportion of animal studies conducted after regulatory approval.

Second, although we identified approved and failed DMTs through various sources, including reviews, national MS treatment guidelines, and a pharmacological database, we acknowledge the potential presence of selection bias in DMT selection. Additionally, another source of potential selection bias is that we manually screened only around half of the full reference library, with the aid of machine learning methods. However, single screening of all unseen records did not identify additionally eligible articles.

Third, we pooled studies with different methodological backgrounds, e.g., using different types of EAE models such as active EAE or adoptive transfer EAE, which is also reflected by a substantial heterogeneity of our analyses.

Fourth, we did not consider conference abstracts for DMT development, which might have mitigated certain biases. Furthermore, we were unable to include unpublished drug testing data from the pharmaceutical industry in our analysis.

Fifth, our focus was on clinically relevant outcome parameters of animal studies, such as clinical disability and neuroimaging outcomes. Hence, we did not consider other commonly used animal study outcome parameters which could be relevant to inform clinical trials, e.g., histopathological, or other behavioural outcomes. In addition, it might be costly for laboratories to implement all these outcomes for assessing putative drug candidates. Nevertheless, this approach enhanced consistency of our dataset.

### Study strengths

The main strength of our study lies in the rigorous systematic review approach used to assess our research objective. This methodology enabled us to reduce various biases commonly associated with such analyses and allowed us to include a relatively large number of both approved and failed DMTs. Additionally, the robustness of our main findings is supported by sensitivity analyses. Together, these factors make our study among the most comprehensive analyses aimed at better understanding animal-to-human drug translation.

### Conclusions

Our systematic review and meta-analysis highlight several key challenges in bridging the gap between laboratory research and real-world clinical application, among them 1) The lack of translational validity of even seemingly clinically relevant outcomes in animal studies, 2) A small repertoire of employed experimental practices, and 3) A presumable disconnect between preclinical and clinical research. To make animal research more valuable and to optimise treatment development for MS, we advocate for fostering closer ties between bench and clinical researchers. Finally, concerted efforts to make preclinical multiple sclerosis drug development more efficient are warranted.

## Contributors

Conception and design of study: IB, PH, WEZ, MR, DSR, BVI; acquisition of data: IB, PH, WEZ, MR, BVI; analysis of data: IB, MR, BVI; drafting the initial manuscript: IB, MR, BVI; data visualization: MR; all authors critically revised the paper draft. All authors read and approved the final version of the manuscript.

## Data sharing statement

All data and code that support the findings of this study are available at our Github: https://github.com/Ineichen-Group/HERMES-MS and from the corresponding author, BVI, upon reasonable request.

## Declaration of interests

The authors report no competing interests related to this study.
