# Supplementary material for: Which experimental factors govern successful animal-to-human translation in multiple sclerosis drug development? A systematic review and meta-analysis
Source: eBioMedicine. 2024 Nov 7;110:105434. doi: 10.1016/j.ebiom.2024.105434 (PMC11582441; doi:10.1016/j.ebiom.2024.105434)
Supplement: Supplementary Figures and Tables [file mmc1.docx]

**Which experimental factors govern successful animal-to-human translation in multiple sclerosis drug development? systematic review and meta-analysis**

Ingrid Berg^1,2^, MSc; Pia Härvelid^1,2^, BSc; Wolfgang E. Zürrer^1,2^, BSc; Marianna Rosso^1^, PhD; Daniel S. Reich^3^, MD, PhD; Benjamin V. Ineichen^1,2^, MD, PhD

**Author affiliations:**

^1^Center for Reproducible Science, University of Zurich, Zurich, Switzerland

^2^Clinical Neuroscience Center, University of Zurich, Switzerland

^3^National Institute of Neurological Disorders and Stroke, National Institutes of Health, Bethesda, MD, USA.

**Correspondence to**:

Benjamin Victor Ineichen, University of Zurich, Center for Reproducible Science, Zurich, Switzerland, ORCID: 0000-0003-1362-4819: [benjamin.ineichen@uzh.ch](mailto:benjamin.ineichen@uzh.ch)

**Supplementary data**

**Supplementary figures**

**Supplementary Figure 1**: Study flow chart.


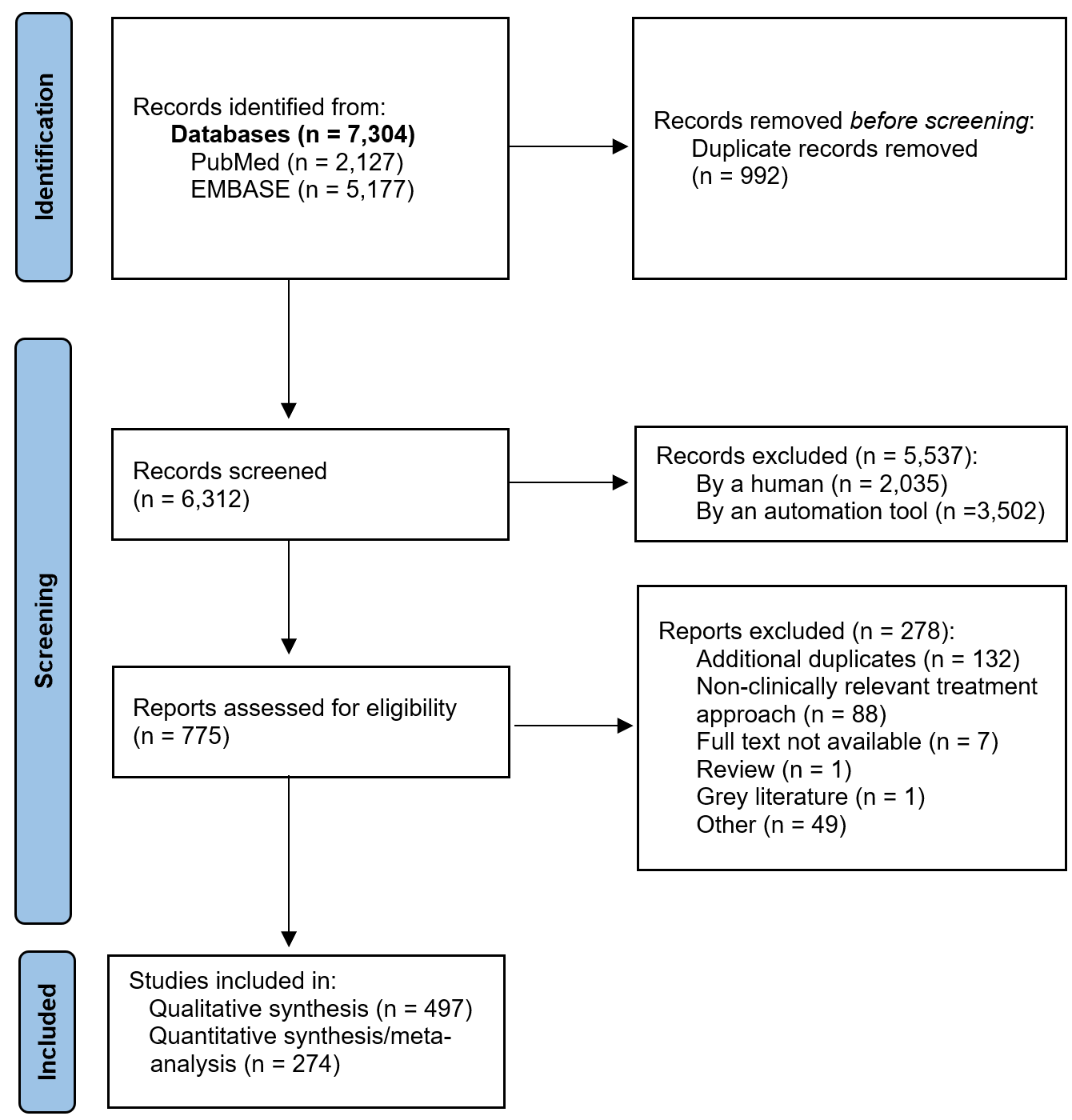


**Supplementary Figure 2**: Risk of bias assessment summary for all included studies.


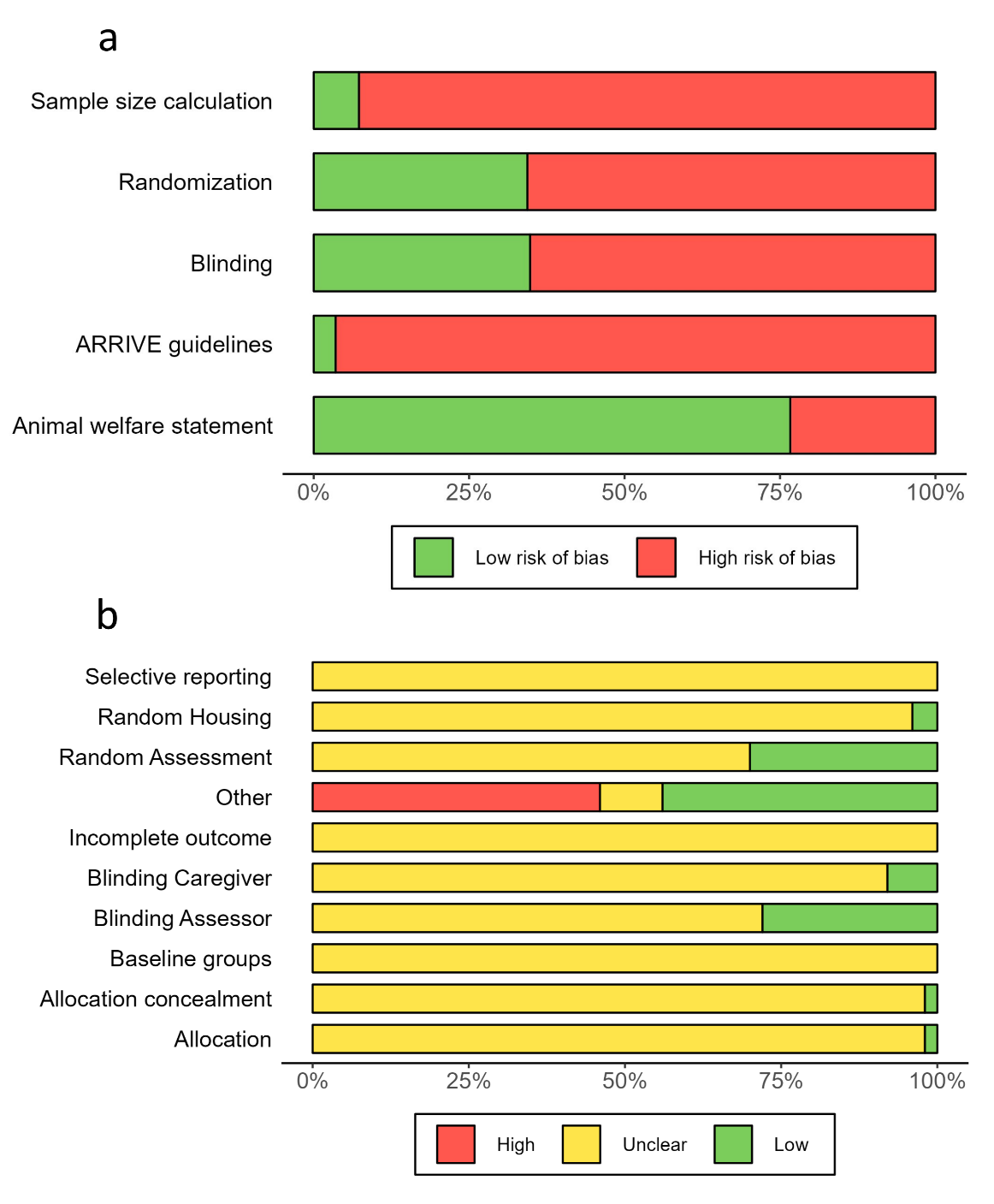


a: The automated risk of bias assessment indicated a low risk of bias in the animal welfare statement domain, moderate bias in the randomization/blinding domains, and a high risk of bias concerning the ARRIVE guidelines and sample size calculation.

b: The SYRCLE risk of bias assessment^1^ suggested unclear risk of bias for most domains, expect for presence of a potential conflict of interest (“Other”) with most studies either having high or low risk of bias. Some studies also showed low risk of bias for blinded experimental assessors and random allocation.

**Supplementary Figure 3**: Sensitivity analysis on effect sizes for EAE scores and MRI outcomes for approved and failed disease-modifying therapies (DMTs).


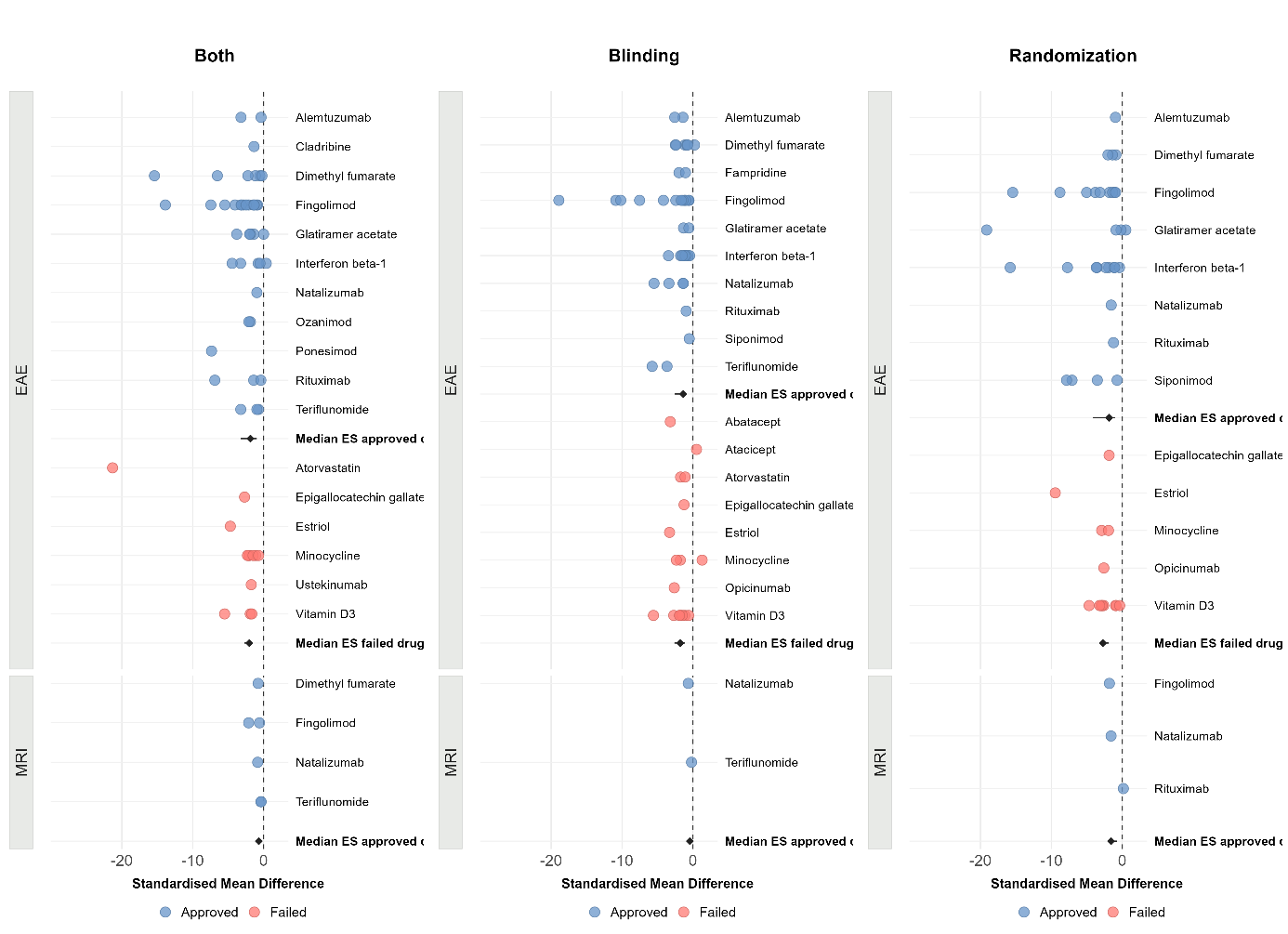


Hedges’ g standardised mean differences per DMT and study and pooled for both EAE scores (top panel) and MRI outcomes (bottom panel), only for animal studies reporting blinding and/or randomization. Pooled effect sizes for approved and failed DMTs are similar.

*Abbreviations: EAE, experimental autoimmune encephalomyelitis; ES, effect sizes; MRI, magnetic resonance imaging.*

**Supplementary Figure 4**: Study countries


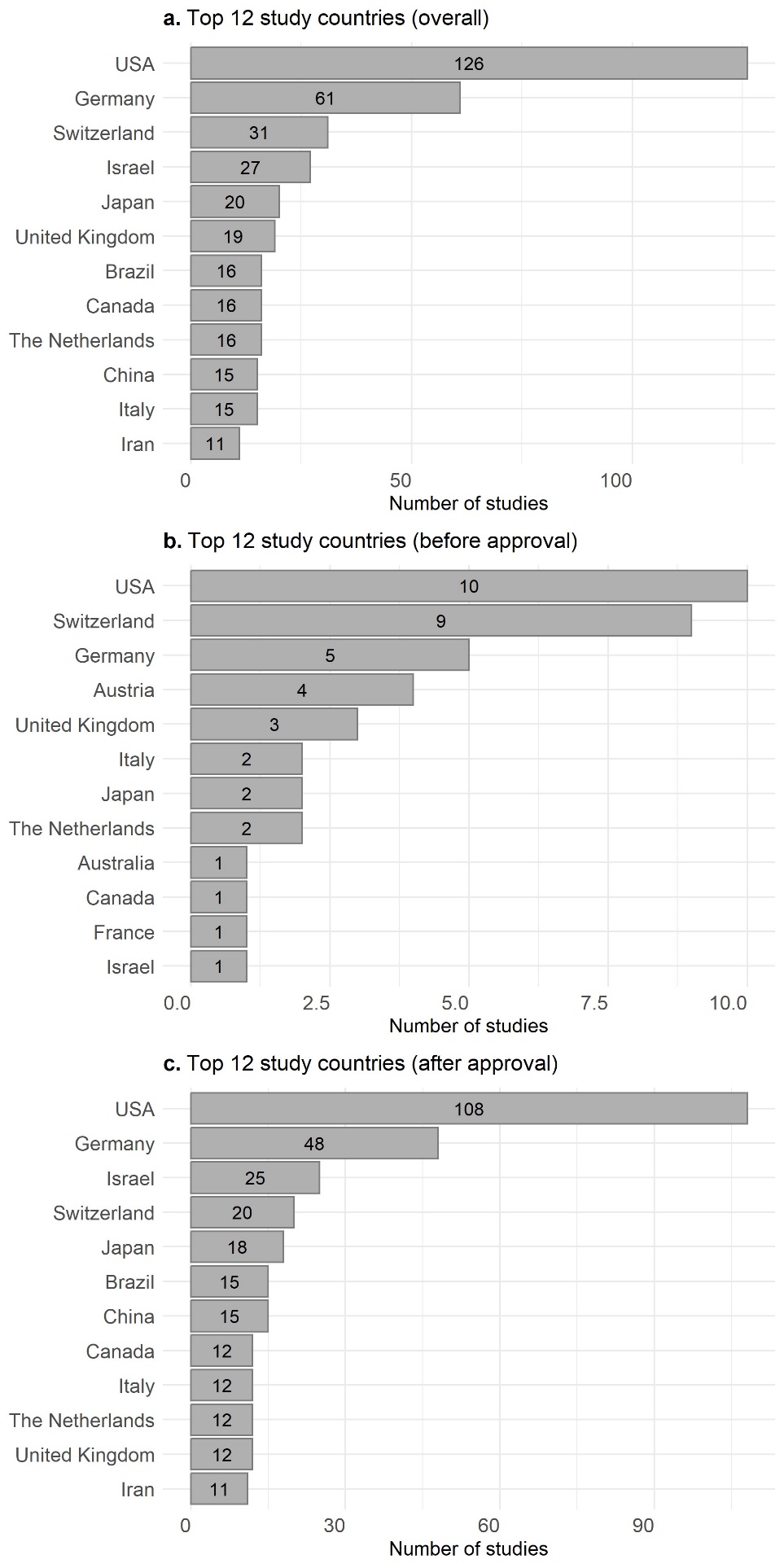


Count of studies testing regulatory-approved disease-modifying therapies (DMTs) in multiple sclerosis animal models overall (a), before regulatory approval (b), and after regulatory approval (c).

**Supplementary tables**

**Supplementary Table 1:** Performance metrics of automated risk of bias assessment.

| **Item** | **FN** | **FP** | **TN** | **TP** | **Sensitivity** | **Specificity** | **Precision** | **F1-score** | **Accuracy** |
| --- | --- | --- | --- | --- | --- | --- | --- | --- | --- |
| Randomization | 2 | 1 | 36 | 14 | 0.88 | 0.97 | 0.93 | **0.90** | 0.94 |
| Blinding | 4 | 0 | 33 | 16 | 0.80 | 1.00 | 1.00 | **0.89** | 0.92 |
| Welfare | 5 | 1 | 10 | 37 | 0.88 | 0.91 | 0.97 | **0.93** | 0.89 |
| Conflict | 0 | 22 | 24 | 7 | 1.00 | 0.52 | 0.24 | 0.39 | 0.58 |
| Sample size | 0 | 1 | 51 | 1 | 1.00 | 0.98 | 0.50 | 0.67 | 0.98 |
| ARRIVE | 0 | 0 | 51 | 2 | 1.00 | 1.00 | 1.00 | **1.00** | 1.00 |

Performance metrics were derived from a manual risk-of-bias assessment of a random subset comprising 53 publications. Our automated extraction tool, developed in the R programming environment^2^, achieved F1-scores exceeding 80% for the majority of items (bold font). Due to subpar performance, we omitted the automated assessment of conflict of interest.

*Abbreviations: FN: false negative FP: false positive, TN: true negative, TP: true positive*

**Supplementary Table 2**: Meta-analysis of MRI outcomes of approved DMTs.

| **Drug** | **Number of studies** | **Effect sizes [95%-CI] (random effects model)** | **Heterogeneity I^2^** | **Number of studies missing (trim-and-fill)** |
| --- | --- | --- | --- | --- |
| **Approved** | | | | |
| Fingolimod | 4 | -10.5 [-18.5 – -2.4] | 97% | 1 |
| Natalizumab | 3 | -3.2 [-6.1 - -0.2] | 92% | 0 |
| **Total** | **7** | **-5.9 [-9.0 – -2.8]** | **95%** | **1** |

One meta-analysis was conducted per DMT and only for DMTs for which at least three studies reported MRI outcomes. Only approved DMTs were available for this analysis. Data presented is Hedges' g standardized mean difference (SMD) and the I^2^ as a measure of heterogeneity. Number of missing studies is presented as computed via trim-and-fill analyses.

**Supplementary Table 3**: Experimental characteristics of animal studies per disease-modifying therapy (DMT).

| **Disease-modifying therapy (DMT)** | **Approval status** | **Number of studies** | **N mean per study** | **Two or more MS models** | **Both sexes** | **Therapeutic regimen** | **Two or more species** | **Two or more strains** | **Two or more outcomes** | **Two or more laboratories** | **Proportion beneficial studies** |
| --- | --- | --- | --- | --- | --- | --- | --- | --- | --- | --- | --- |
| Alemtuzumab | Approved | 11 | 55.50 | No (EAE) | Yes | Yes | No (mouse) | Yes (C57BL, SJL) | No | Yes | 0.82 |
| Cladribine | Approved | 1 | NR | No (EAE) | No | No | No (mouse) | No (C57BL) | No | Yes | 1.00 |
| Dimethyl fumarate | Approved | 36 | 41.75 | Yes (EAE, cuprizone, TMEV) | Yes | Yes | Yes (mouse, rat) | Yes (C57BL, SJL, DA, Lewis, Wistar) | No | Yes | 0.816 |
| Fampridine | Approved | 10 | 48.00 | Yes (EAE, cuprizone, EtBr) | Yes | Yes | Yes (mouse, rat) | Yes (C57BL, SJL, Lewis, Wistar, SD) | Yes | Yes | 0.50 |
| Fingolimod | Approved | 97 | 63.80 | Yes (EAE, cuprizone, lysolecithin, TMEV, EtBr | Yes | Yes | Yes (mouse, rat, zebrafish) | Yes (C57BL, SJL, Biozzi, Lewis, DA, Wistar, SD, BNy) | Yes | Yes | 0.87 |
| Glatiramer acetate | Approved | 72 | 56.15 | Yes (EAE, TMEV, cuprizone, lysolecithin, OSE) | Yes | Yes | Yes (mouse, rat) | Yes (C57BL, SJL, Biozzi, Lewis, DA, Wistar, SD, BN) | Yes | Yes | 0.92 |
| Interferon beta-1 | Approved | 64 | 55.58 | Yes (EAE, EtBr, TMEV) | Yes | Yes | Yes (mouse, rat, guinea pig) | Yes (C57BL, SJL, Biozzi, Lewis, Wistar, SD, BN, Hartley) | No | Yes | 0.72 |
| Monomethyl fumarate | Approved | 2 | NR | No (EAE) | No | No | No (mouse) | No (SJL) | No | Yes | 1.00 |
| Natalizumab | Approved | 16 | 47.50 | No (EAE) | Yes | Yes | Yes (mouse, rat, guinea pig, monkey) | Yes (C57BL, SJL, Lewis, DA, Hartley, Rhesus) | Yes | Yes | 0.75 |
| Ozanimod | Approved | 3 | 67.00 | No (EAE) | No | Yes | No (mouse) | No (C57BL/6) | No | Yes | 1.00 |
| Pefinterferon beta-1a | Approved | 1 | 80.00 | No (TMEV) | No | Yes | No (mouse) | No (SJL) | No | Yes | 1.00 |
| Ponesimod | Approved | 4 | 71.00 | Yes (EAE, cuprizone) | Yes | Yes | No (mouse) | No (C57BL/6) | No | Yes | 1.00 |
| Rituximab | Approved | 17 | 14.00 | No (EAE) | Yes | Yes | Yes (mouse, rat, marmoset) | Yes (C57BL/6, SJL, Lewis) | Yes | Yes | 0.823529 |
| Siponimod | Approved | 13 | 90.00 | Yes (EAE, cuprizone, TMEV) | Yes | Yes | Yes (mouse, rat) | Yes (C57BL/6, SJL, DA) | Yes | Yes | 1.00 |
| Teriflunomide | Approved | 12 | 89.50 | Yes (EAE, cuprizone, TMEV, lysolecithin) | Yes | Yes | Yes (mouse, rat, xenopus) | Yes (C57BL/6, SJL, DA, Wistar) | Yes | Yes | 0.75 |
| Abatacept | Failed | 12 | 53.00 | No (EAE) | No | Yes | Yes (mouse, rat) | Yes (C57BL/6, SJL, Biozzi, Lewis) | No | Yes | 0.67 |
| Atacicept | Failed | 1 | 57.00 | No (EAE) | No | No | No (mouse) | No (BN) | No | Yes | 0 |
| Atorvastatin | Failed | 12 | 34.00 | No (EAE) | Yes | Yes | No (mouse) | Yes (C57BL/6, SJL) | No | Yes | 0.92 |
| Epigallocatechin gallate | Failed | 8 | 54.00 | Yes (EAE, cuprizone) | Yes | Yes | No (mouse) | Yes (C57BL/6, SJL) | No | Yes | 1.00 |
| Estriol | Failed | 18 | 56.17 | No (EAE) | Yes | Yes | No (mouse) | Yes (C57BL/6, SJL) | No | Yes | 1.00 |
| Inosine | Failed | 1 | 20.00 | No (EAE) | No | No | No (mouse) | No (C57BL/6) | No | No | 1.00 |
| Minocycline | Failed | 26 | 46.75 | Yes (EAE, EtBr, cuprizone) | Yes | Yes | Yes (mouse, rat) | Yes (C57BL/6, SJL, Lewis, DA, Wistar, SD) | No | Yes | 0.73 |
| Opicinumab | Failed | 8 | 40.337 | Yes (EAE, cuprizone, lysolecithin) | Yes | Yes | Yes (mouse, rat) | Yes (C57BL/6, SD, BN) | Yes | Yes | 0.75 |
| Toralizumab | Failed | 1 | NR | No (EAE) | No | Yes | No (mouse) | No (SJL) | No | No | 1.00 |
| Ustekinumab | Failed | 1 | NR | No (EAE) | No | Yes | No (mouse) | No (C57BL/6) | No | No | 1.00 |
| Vitamin D3 | Failed | 83 | 75.65 | Yes (EAE, cuprizone, EtBr, lysolecithin) | Yes | Yes | Yes (mouse, rat) | Yes (C57BL/6, SJL, Lewis, DA, Wistar, SD) | Yes | Yes | 0.93 |

Animal testing of approved and failed DMTs with experimental characteristics: Number of animal studies, mean number of animals per study as well as whether they have been tested in two or more MS animal models, both sexes, with a therapeutic regimen (compared to a prophylactic regimen), in two or more species/strains, with two or more outcomes (EAE scores and MRI), in two or more laboratories. The last column presents the mean proportion of animal studies with at least one beneficial outcome.

*Abbreviations: BN, Brown Norway rats; DA, Dark Agouti rats; EAE, experimental autoimmune encephalomyelitis; NR, not reported; OSE, opticospinal EAE; SD, Sprague-Dawley rats; TMEV, Theiler’s murine encephalomyelitis virus.*

**Supplementary Table 4:** Median years and ranges for first-in-animal and first-in-MS trials for approved and failed disease-modifying therapies (DMTs).

| **Approved DMTs** | Median | Range |
| --- | --- | --- |
| first animal trial | 2011 | 1979-2019 |
| first human MS trial | 2006 | 1977-2019 |
| **Failed DMTs** |  |  |
| first animal trial | 2002 | 1991-2016 |
| first human MS trial | 2006 | 1986-2018 |

*Abbreviations: DMT, disease-modifying therapies; MS, multiple sclerosis.*

**Supplementary forest plots**

A. Forest plots of EAE scores for approved and failed DMTs, with at least 3 studies

in alphabetical order.

DMTs with < 3 studies with no forest plot: cladribine, fampridine, monomethyl fumarate, ocrelizumab, ofatumumab, peginterferon beta-1, ponesimod (approved), acyclovir, atacicept, autologous T cell vaccine, BGC-200134, BHT-3009, BX-471, dirucotide, efalizumab, inosine, opicinumab, plozalizumab, rosiglitazone, rovelizumab, tabalumab, temelimab, tiplimotide, toralizumab, ustekinumab, vatelizumab (failed).

**Abatacept (failed)**


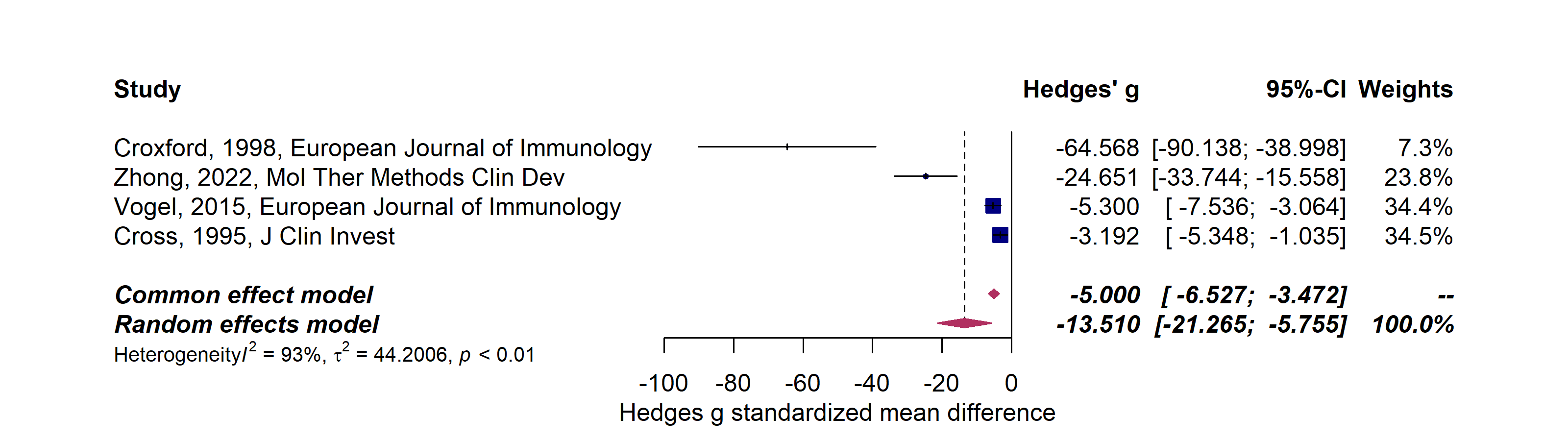


**Alemtuzumab (approved)**


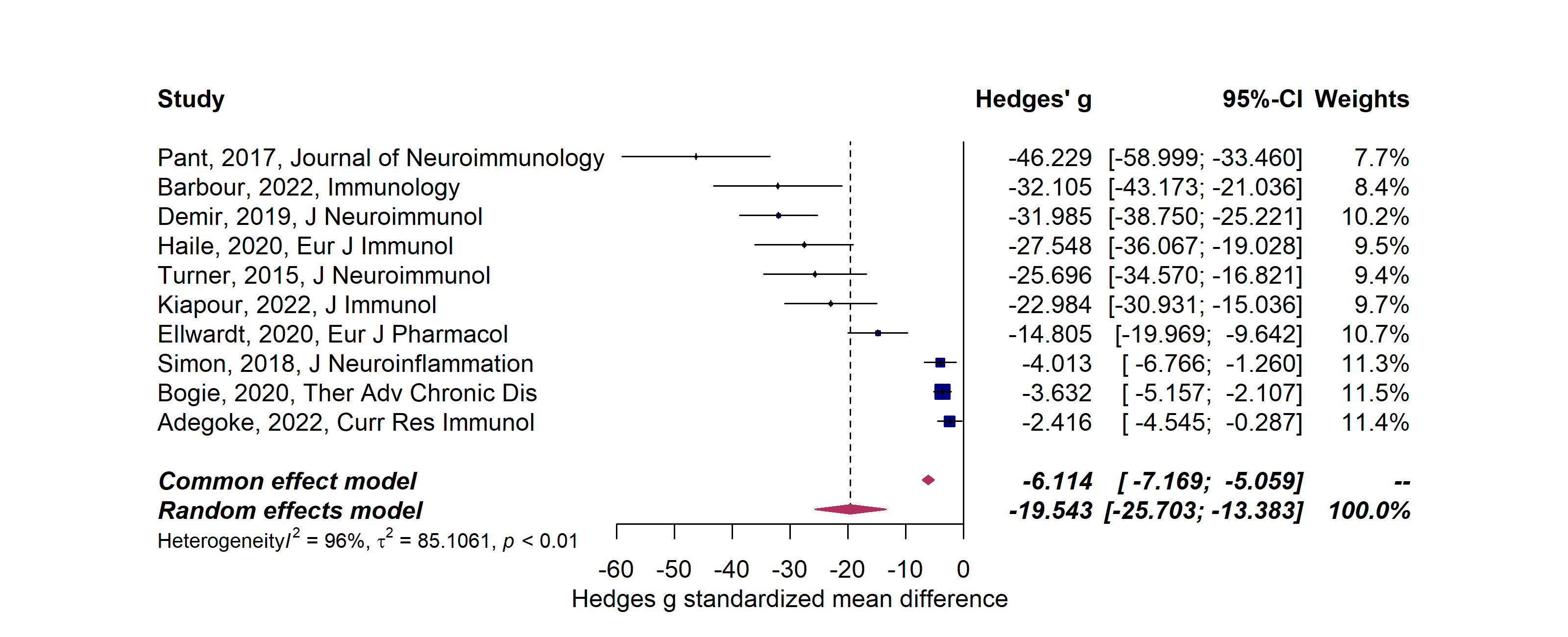


**Atorvastatin (failed)**


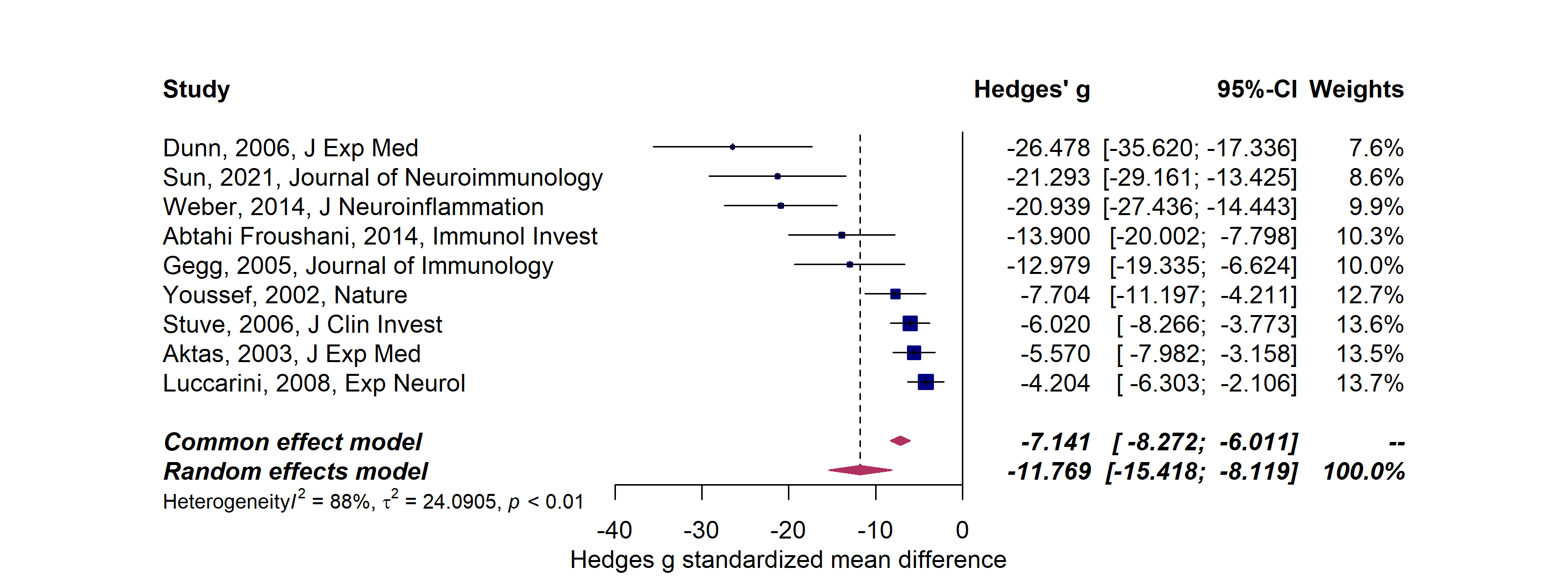


**Vitamin** **D3 (failed)**


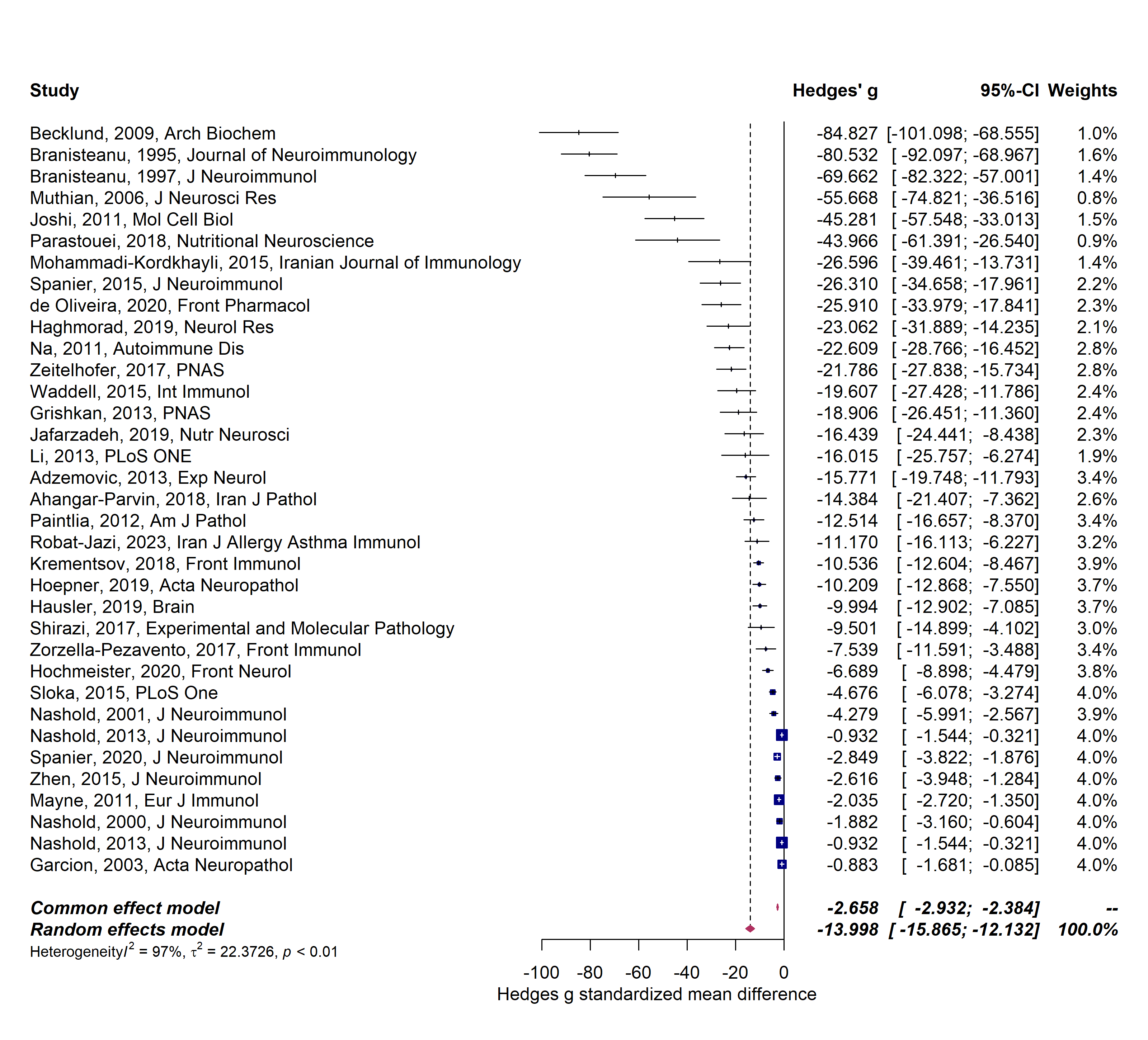


**Dimethyl fumarate (approved)**


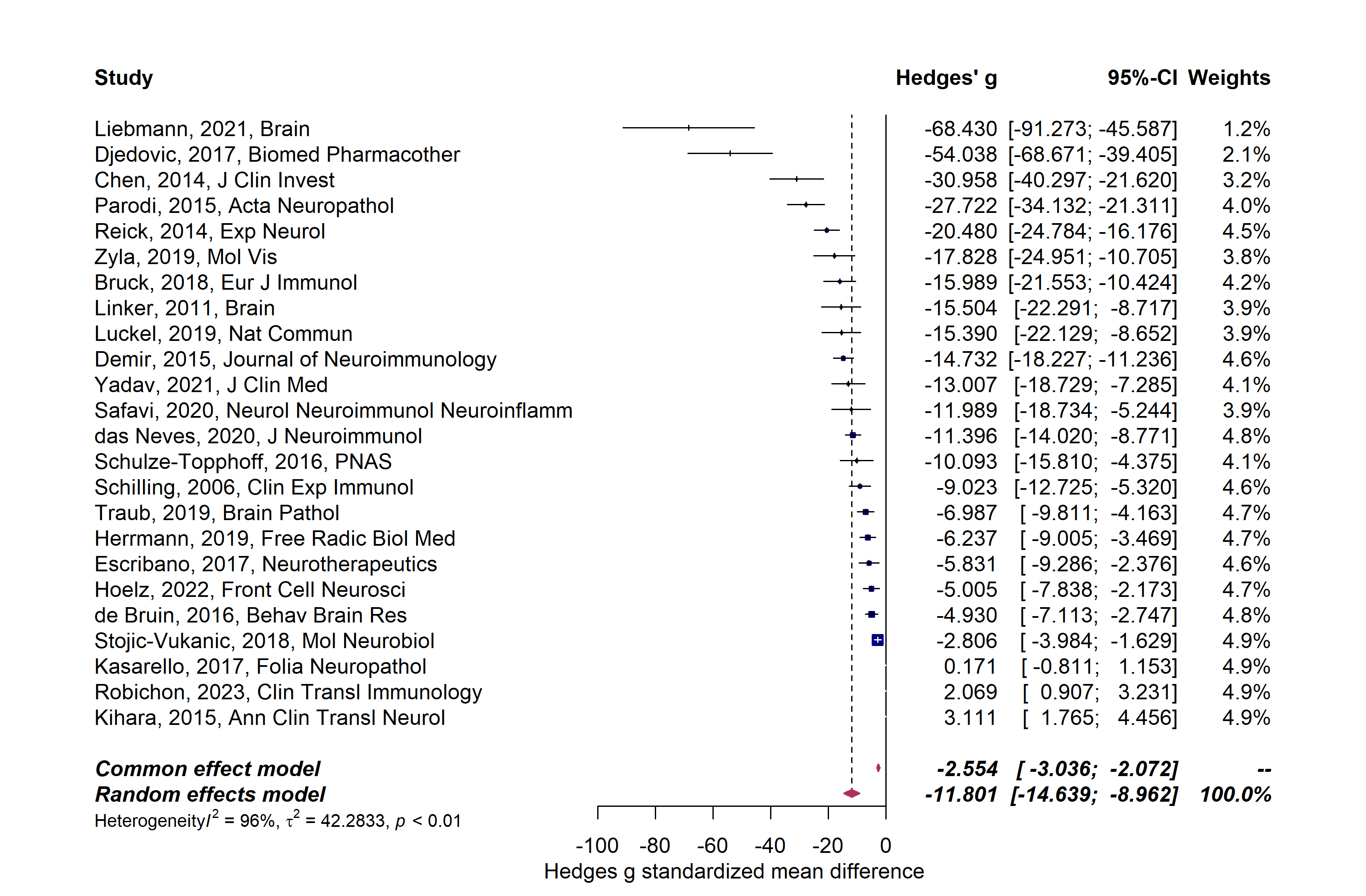


**Epigallocatechin gallate (failed)**


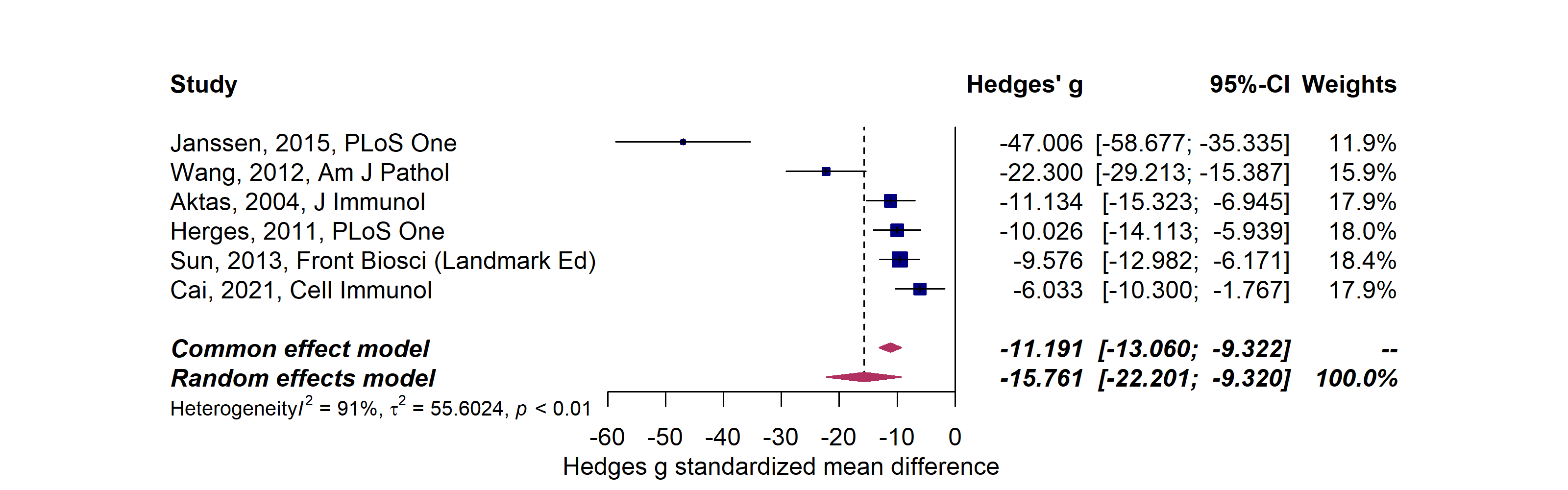


**Estriol (failed)**


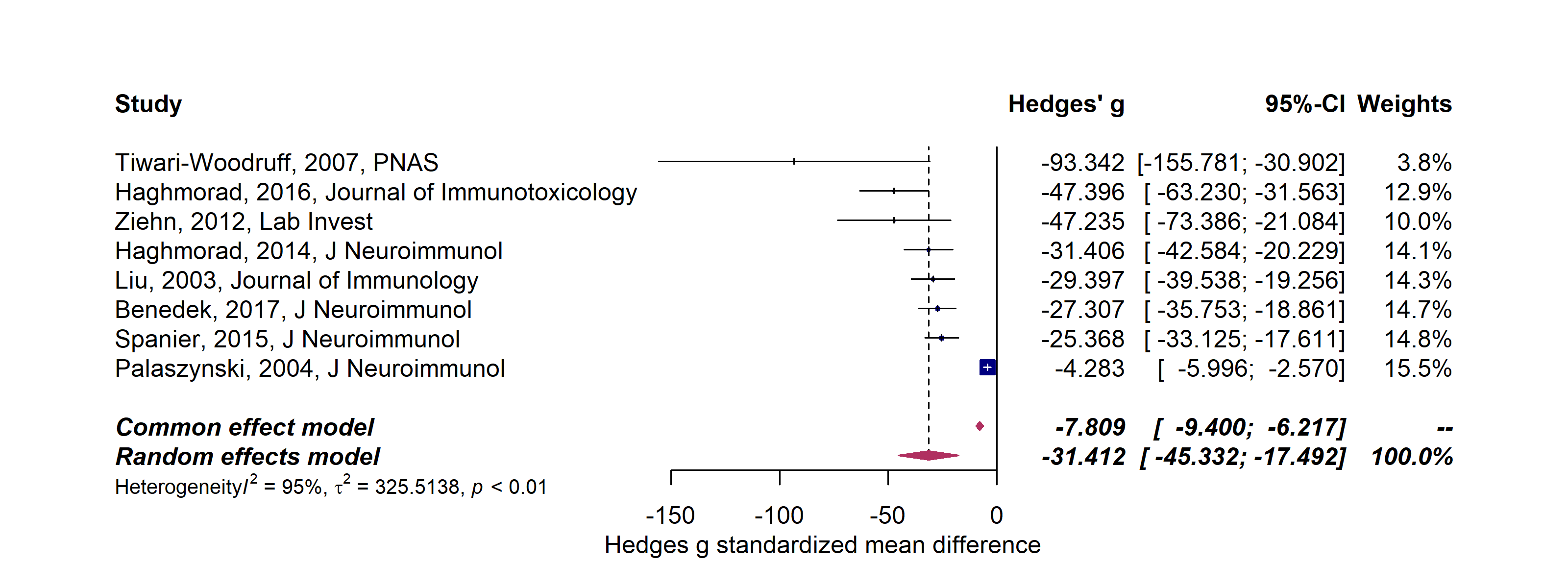


**Fingolimod (approved)**


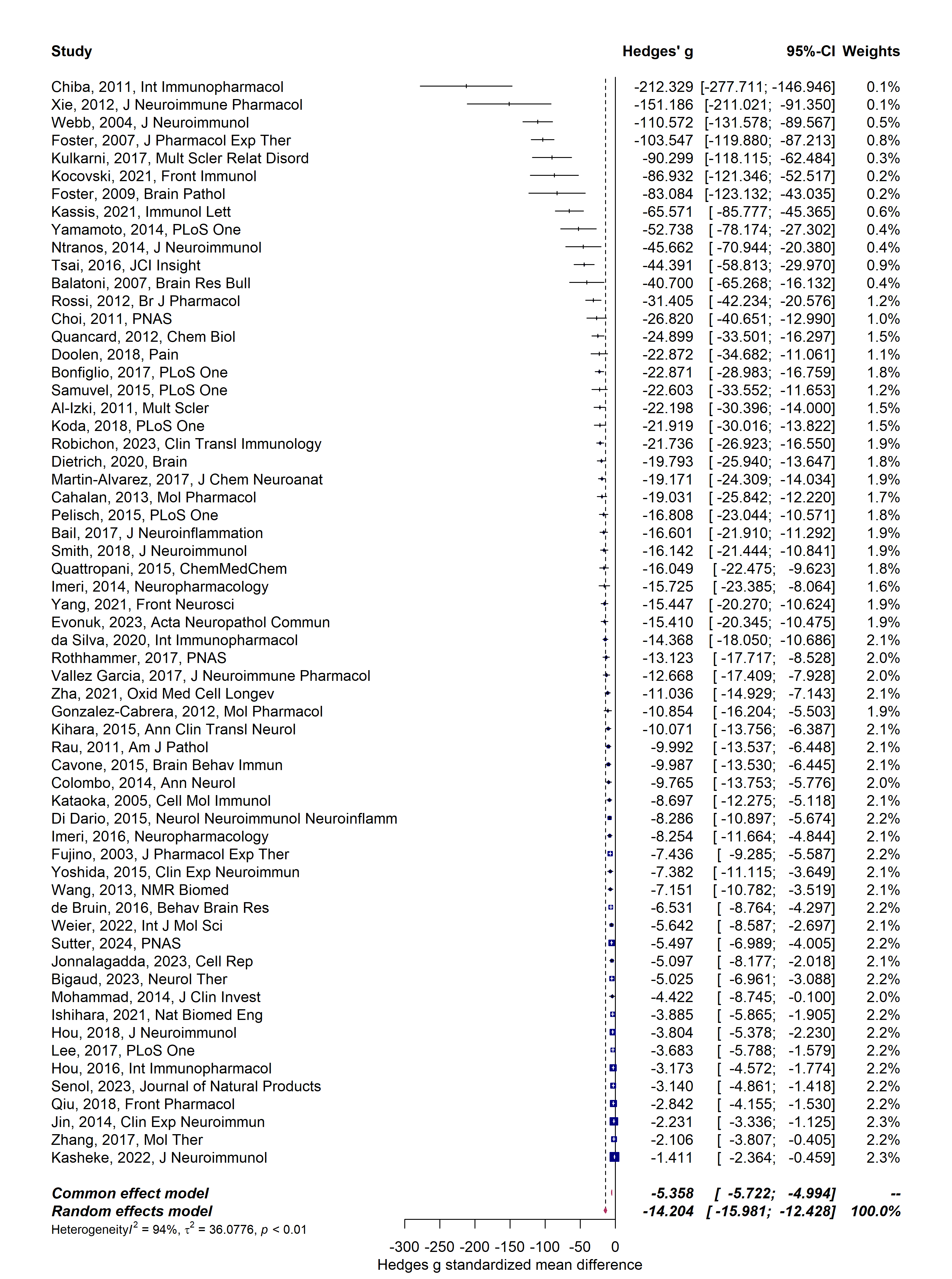


**Glatiramer acetate (approved)**


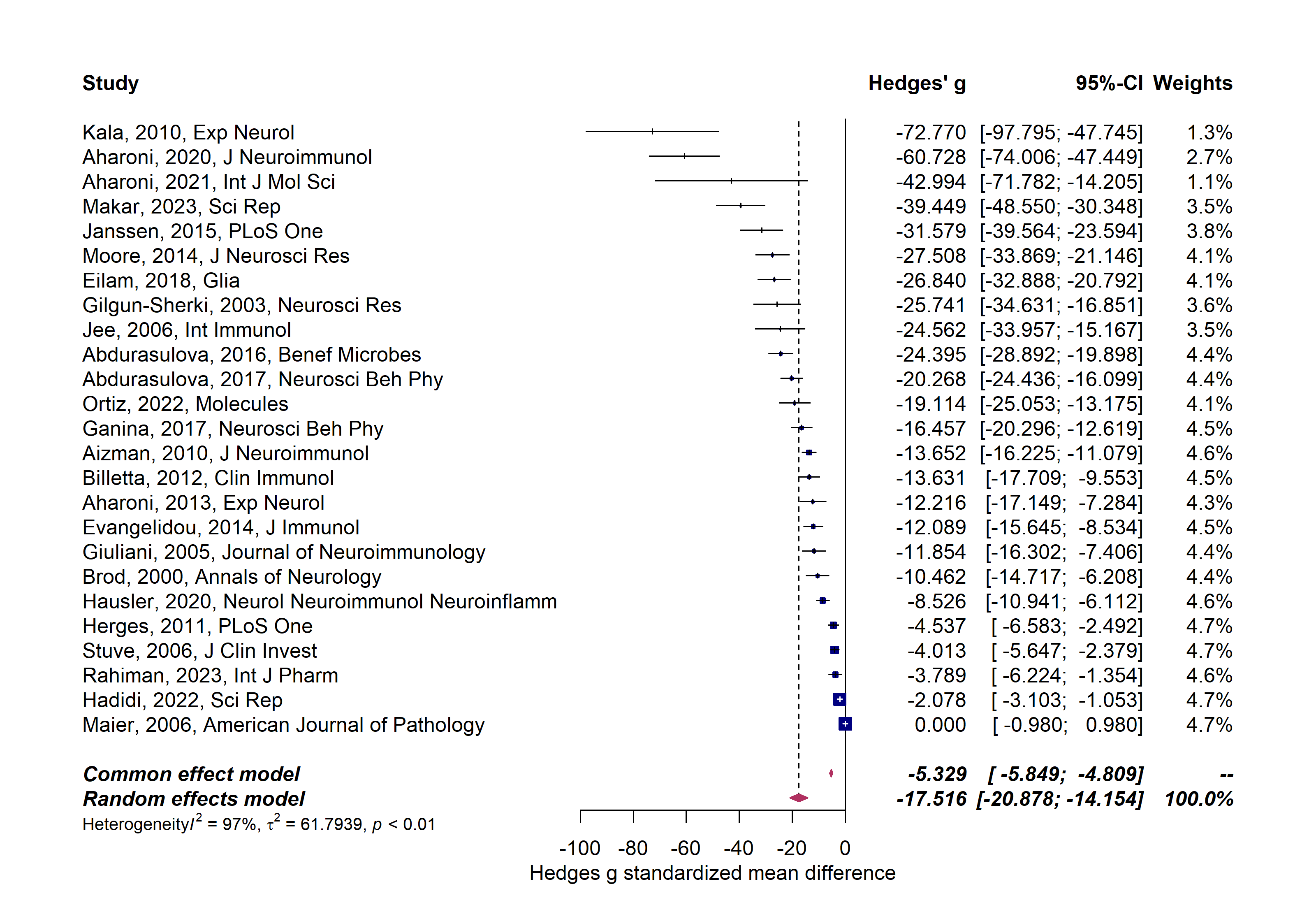


**Interferon Beta (approved)**


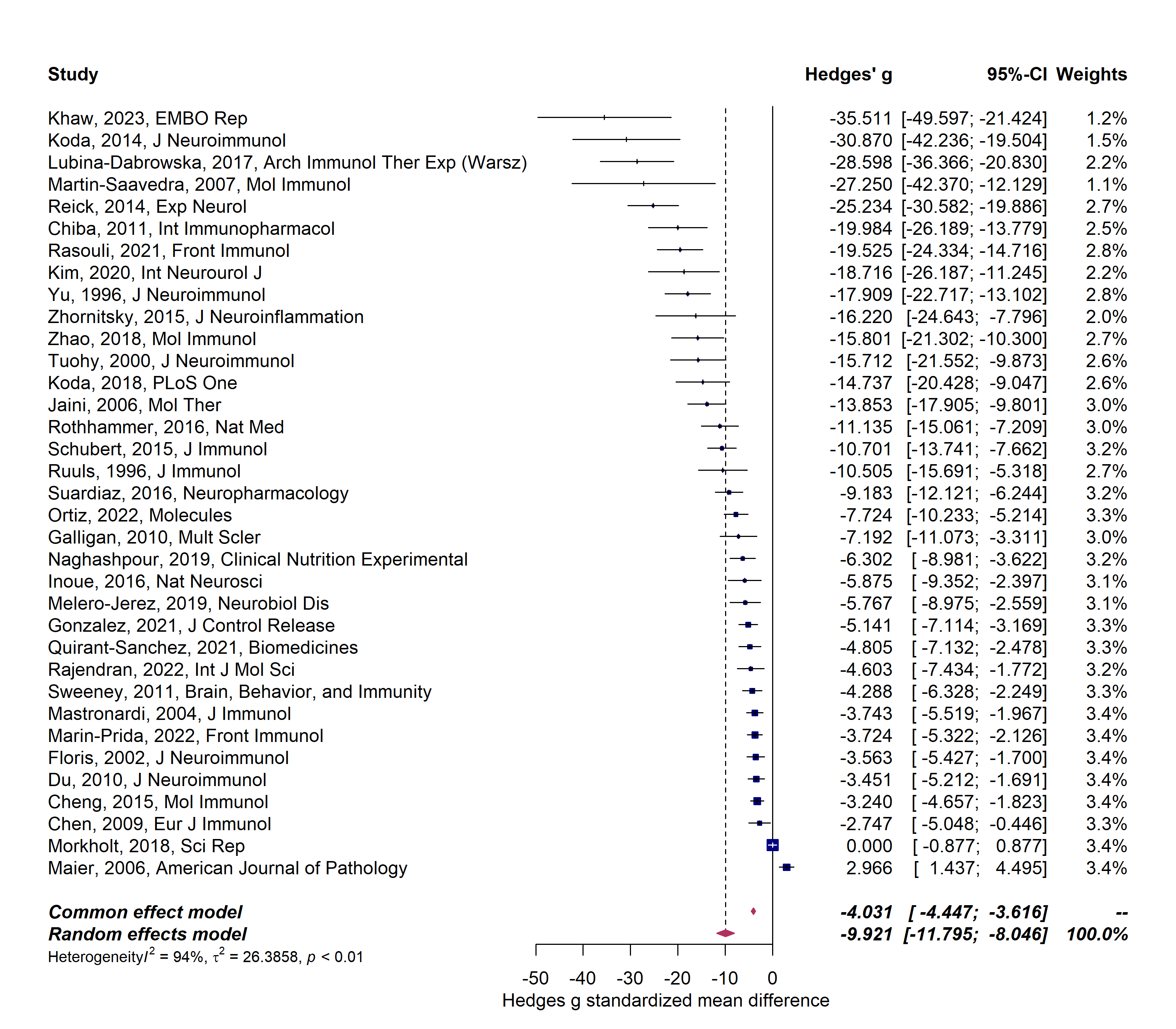


**Ozanimod (approved)**


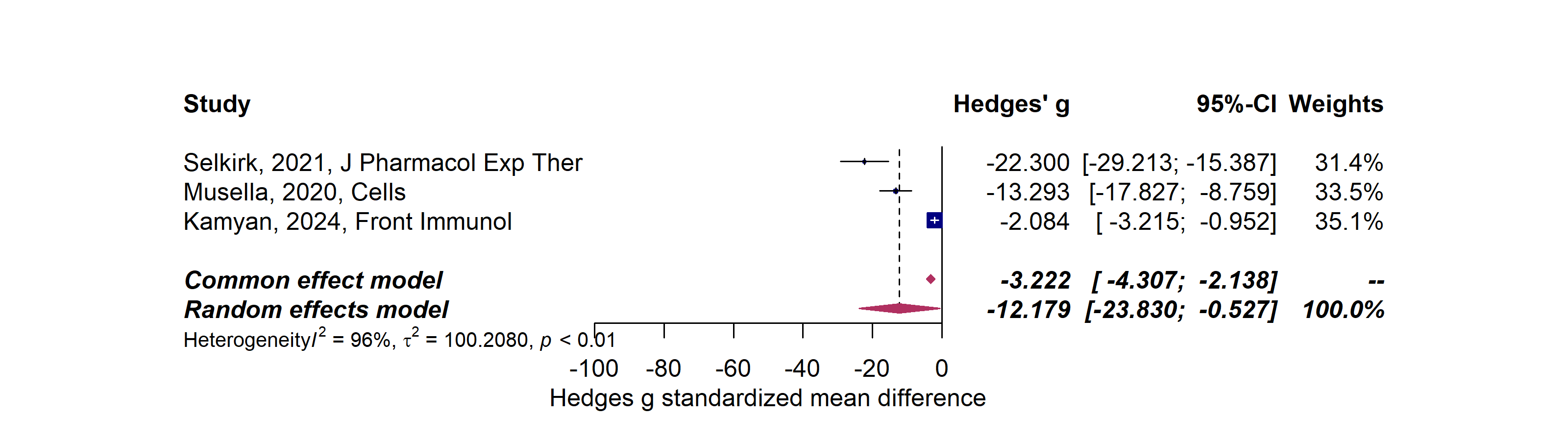


**Minocycline (failed)**


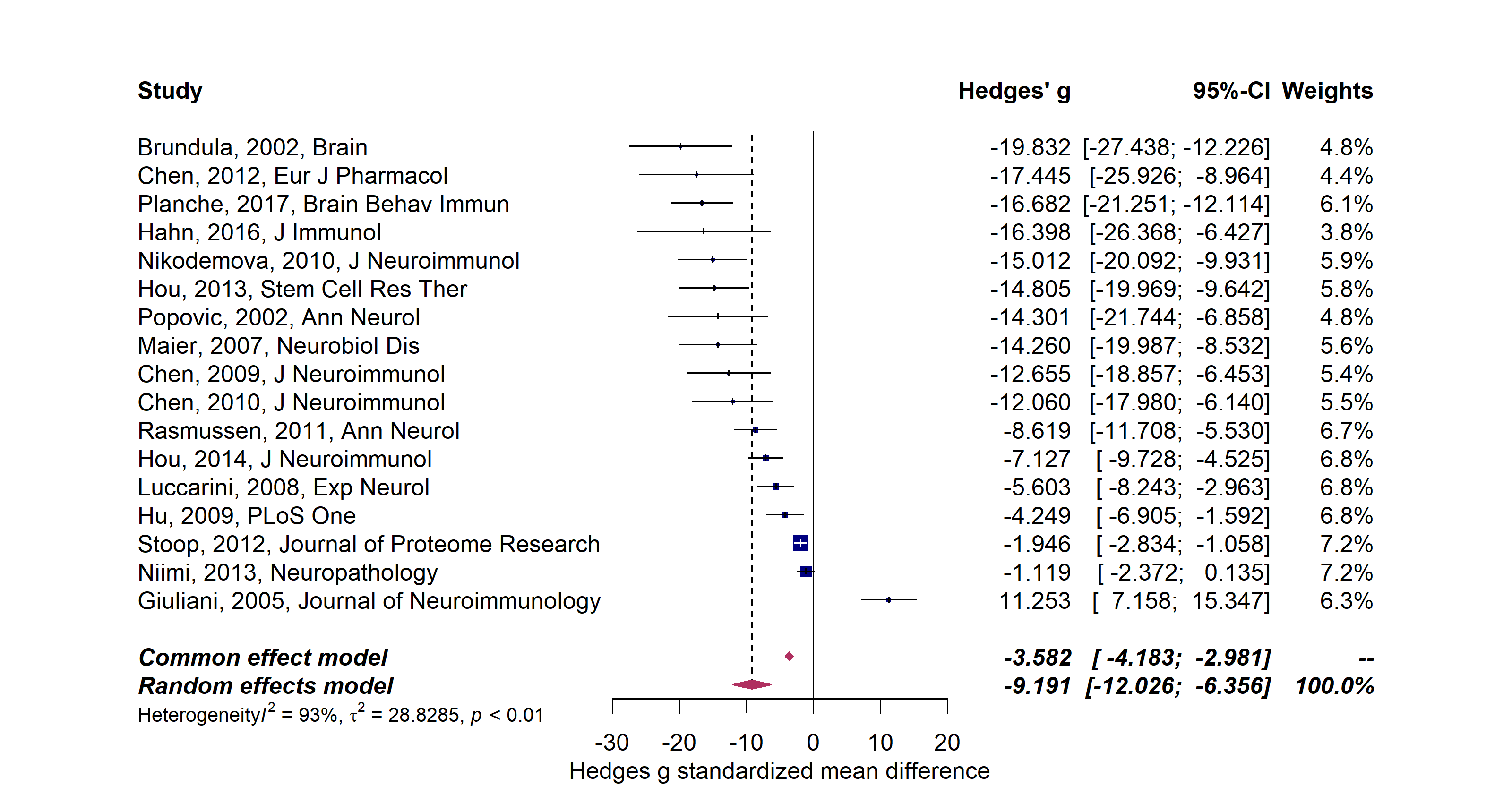


**Natalizumab (approved)**


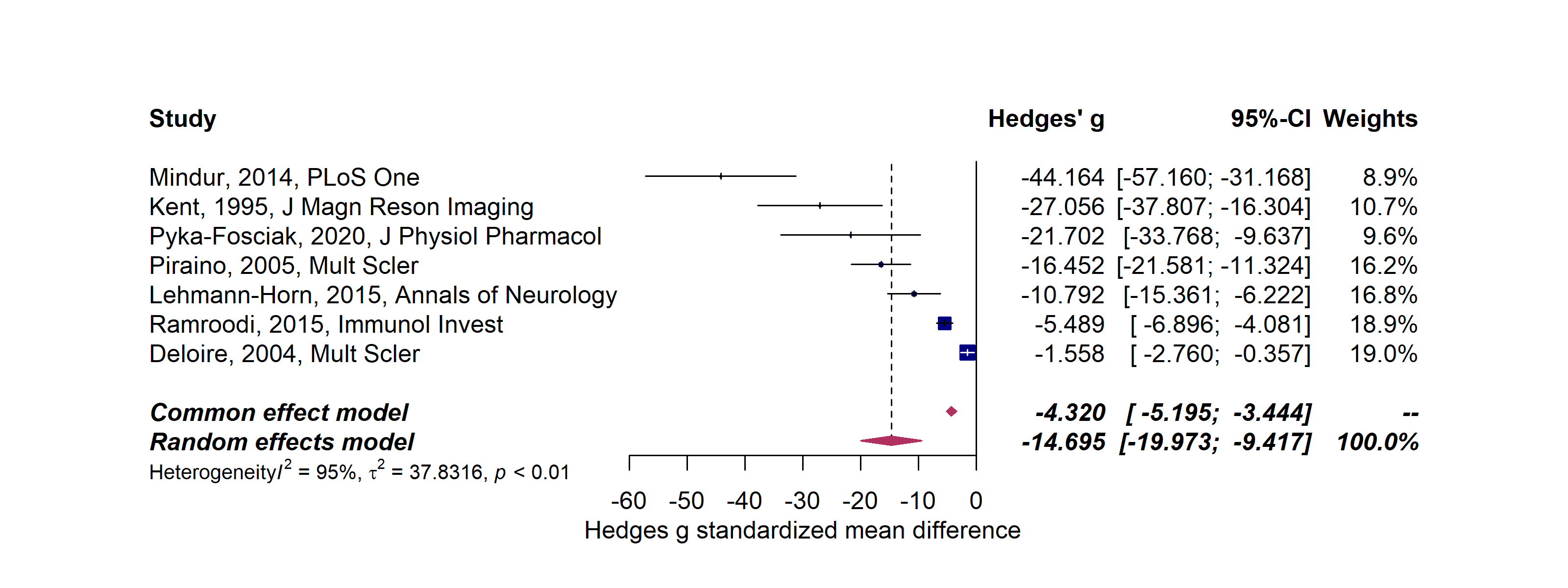


**Rituximab (approved)**


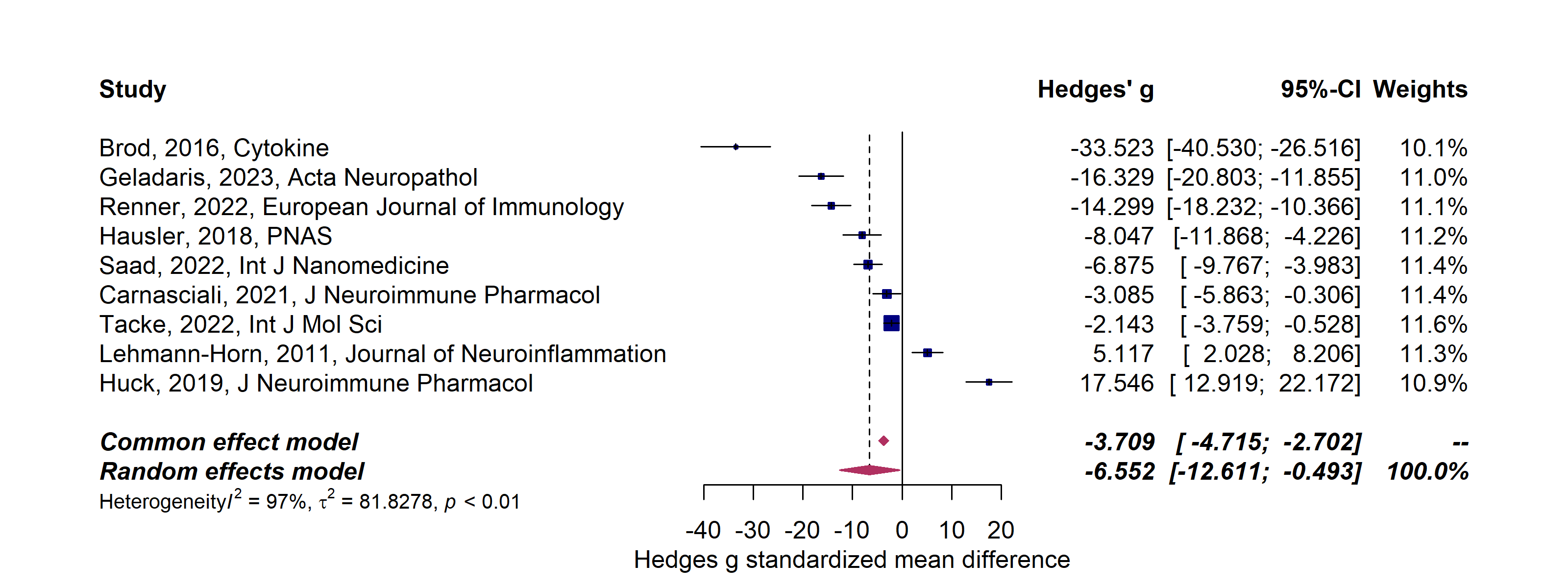


**Siponimod (approved)**


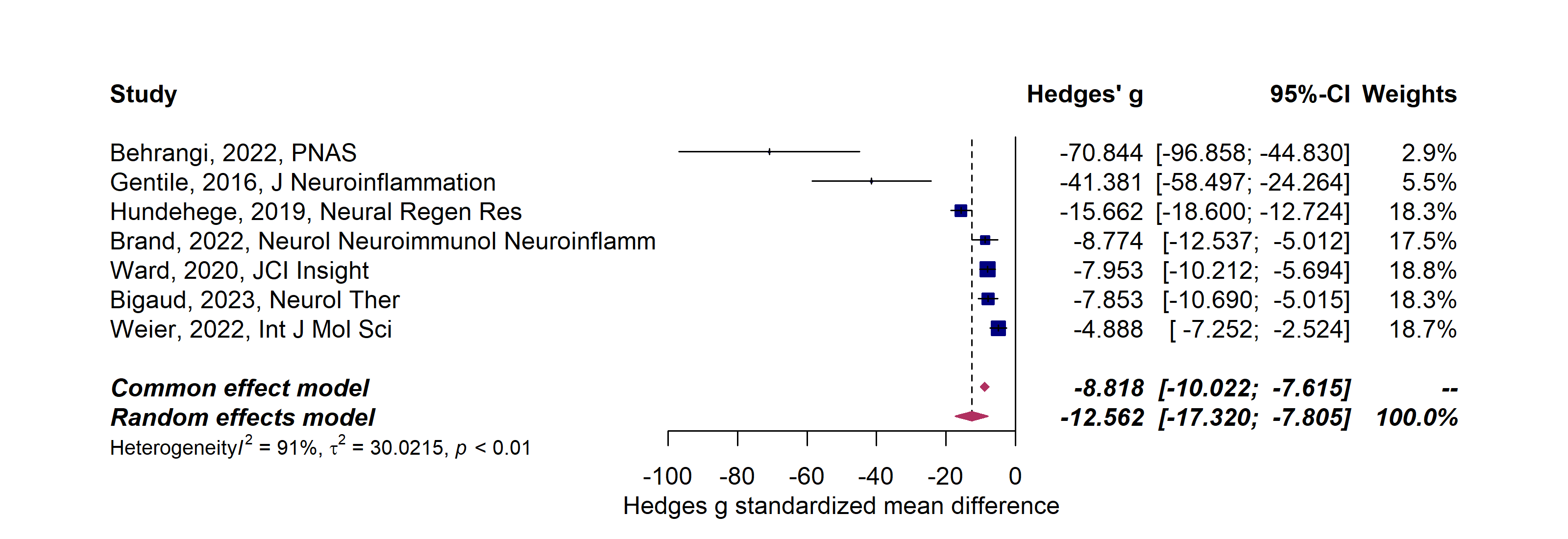


**Teriflunomide (approved)**


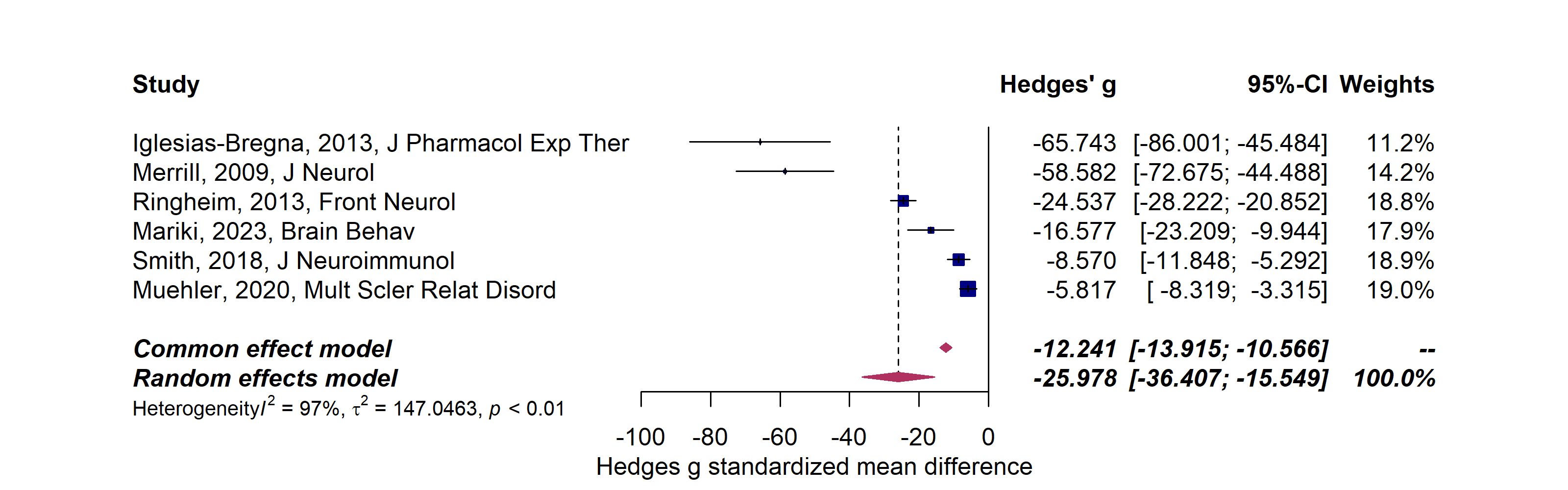


B. Forest plots of MRI outcomes for approved DMTs, with at least 3 studies

in alphabetical order.

DMTs with < 3 studies with no forest plot: alemtuzumab, cladribine, dimethyl fumarate, fampridine, glatiramer acetate, interferon Beta 1, monomethyl fumarate, ocrelizumab, ofatumumab, ozanimod, peginterferon beta-1, ponesimod, rituximab, Siponimod, teriflunomide (approved), abatacept, acyclovir, atacicept, atorvastatin, autologous T cell vaccine, BGC-200134, BHT-3009, BX-471, dirucotide, efalizumab, epigallocatechin gallate, estriol, inosine, minocycline, opicinumab, plozalizumab, rosiglitazone, rovelizumab, tabalumab, temelimab, tiplimotide, toralizumab, ustekinumab, vatelizumab, vitamin D3 (failed).

**Fingolimod MRI (approved)**


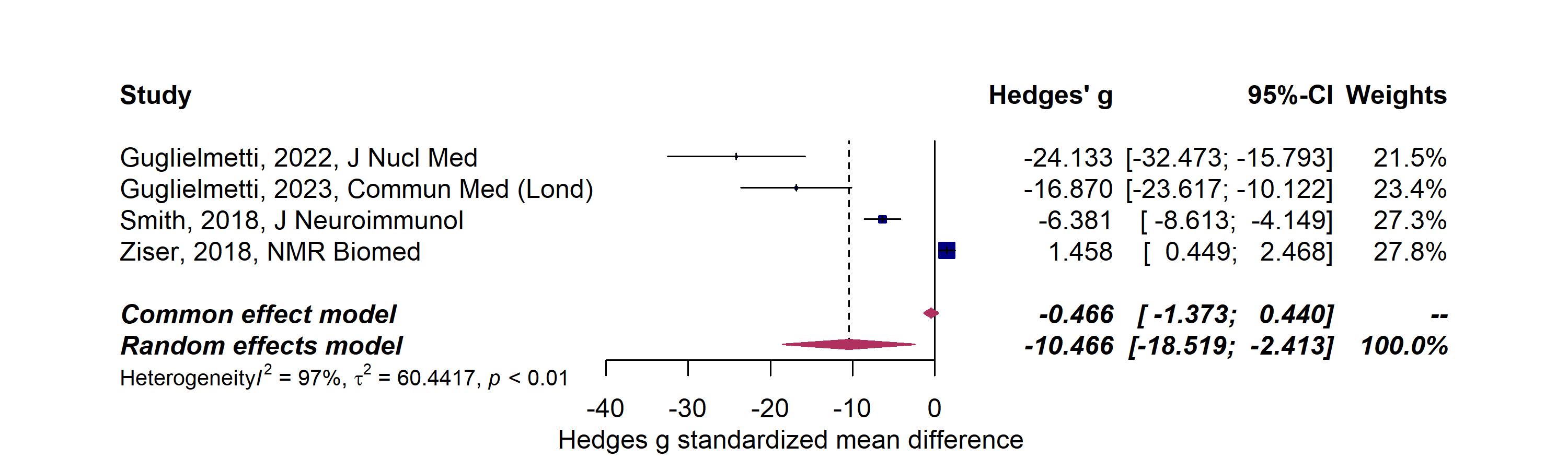


**Natalizumab MRI (approved)**


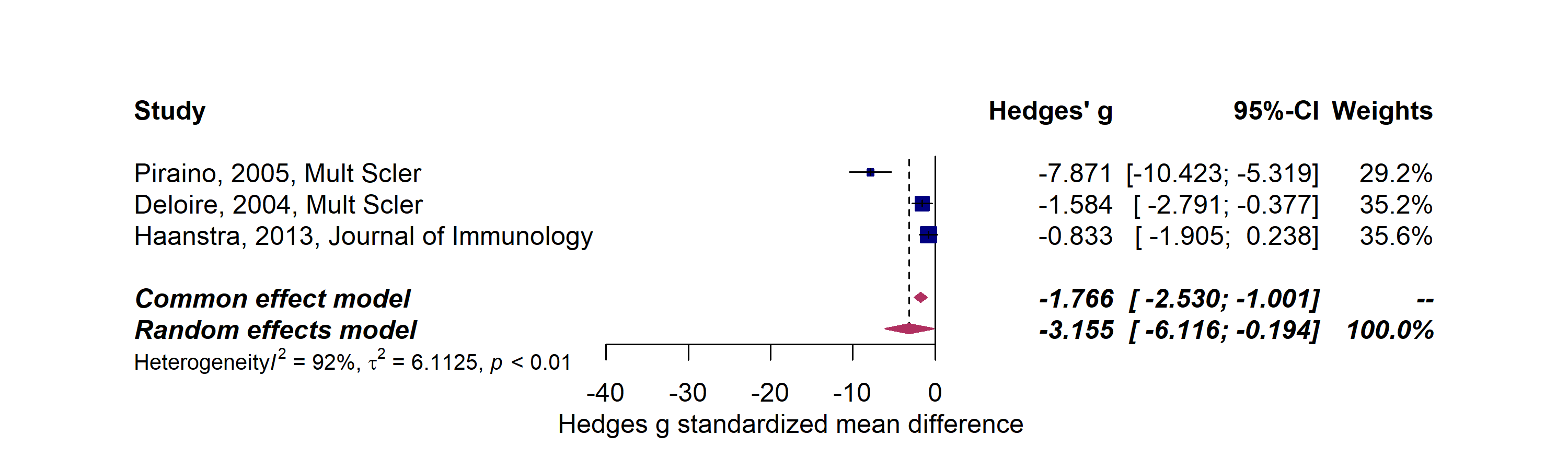


C. Funnel plots


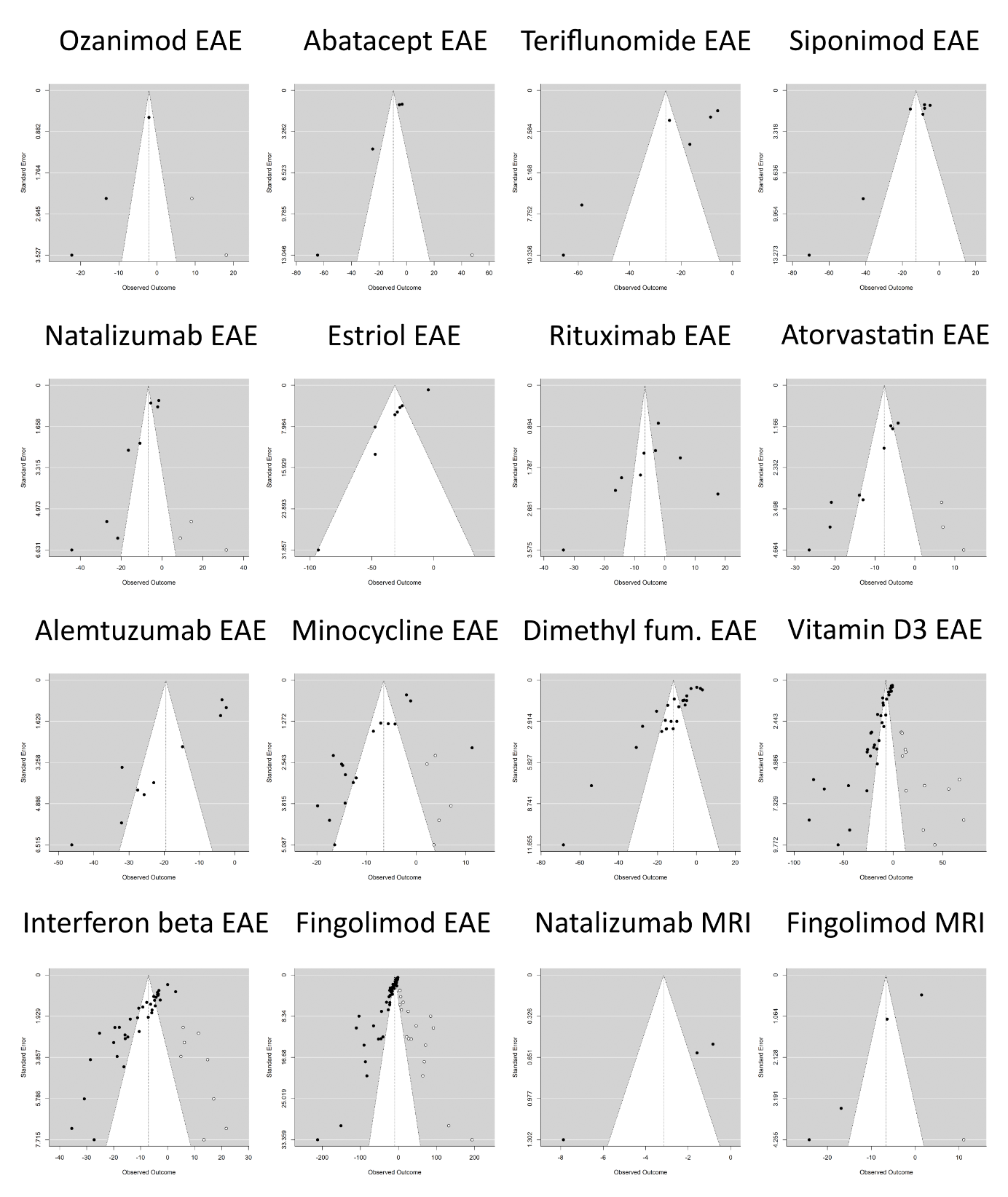


**Search string**

((("abatacept" OR "BMS-188667" OR "BMS 188667" OR "BMS188667" OR "BMS-188667SC" OR "CTLA4-Ig" OR "hCTLA4-Ig-Bristol-Myers-Squibb" OR "ONO-4164" OR "ONO 4164" OR "ONO4164" OR "ONO-4164-IV" OR "ONO-4164-SC" OR "Orencia" OR "CTLA4-IG4m" OR "RG1046" OR "RG-1046" OR "RG 1046" OR "RG2077" OR "RG-2077" OR "RG 2077") OR

("aciclovir" OR "acyclovir" OR "acycloguanosine") OR

("atacicept" OR "TACI-Ig") OR

("atorvastatin" OR "Lipitor") OR

("Imilecleucel-T" OR "Tcelna" OR "Tovaxin") OR

("BGC 200134" OR "BGC-200134" OR "BGC200134" OR "Pleneva") OR

("BHT 3009" OR "BHT-3009" OR "BHT3009" OR "BHT-300904" OR "BHT 300904" OR "BHT300904") OR

("BX 471" OR "BX-471" OR "BX471" OR "BAY 865047" OR "BAY-865047" OR "BAY865047" OR "SH T 04268H" OR "SH T-04268H" OR "SH T04268H" OR "ZK-811752" OR "ZK 811752" OR "ZK811752") OR

("dirucotide" OR "MBP 8298" OR "MBP-8298" OR "MBP8298" OR "SF328" OR "SF 328" OR "SF-328") OR

("Efalizumab" OR "Anti-CD11a monoclonal antibody" OR "HU 1124" OR "Hu1124" OR "Hu-1124" OR "Hu 1124" OR "Raptiva" OR "Xanelim") OR

("epigallocatechin gallate" OR "EGCG" OR "epigallocatechin-3-gallate" OR "Sunphenon") OR

("estriol" OR "oestriol" OR "estratriol" OR "theelol" OR "trihydroxyestrin" OR "trihydroxyoestrin") OR

("inosine" OR "Riboxinum" OR "Runihol" OR "Axosine") OR

("minocycline" OR "Minocin" OR "Minomycin" OR "Akamin" OR "Amzeeq" OR "RPX 602" OR "RPX-602" OR "RPX602" OR "DFD29" OR "DFD-29" OR "DFD 29" OR "HY-02" OR "HY02" OR "HY 02" OR "Meizuvo" OR "Ximino") OR

("opicinumab" OR "BIIB033" OR "BIIB-033" OR "BIIB 033" OR "anti-LINGO" OR "anti-LINGO-1 antibody") OR

("plozalizumab" OR "anti-CCR2 monoclonal antibody" OR "MLN-1202" OR "MLN1202" OR "MLN 1202" OR "TAK-202" OR "TAK 202" OR "TAK202") OR

("raltegravir" OR "Isentress" OR "L 900612" OR "L900612" OR "L-900612" OR "MK-0518" OR "MK 0518" OR "MK0518") OR

("rituximab" OR "GP-2013" OR "GP2013" OR "GP 2013" OR "Rixathon" OR "Riximyo" OR "SDZ-RTX" OR "BX 2336" OR "BX-2336" OR "BX2336" OR "BXT-2336" OR "BXT2336" OR "BXT 2336" OR "Novex" OR "Rituxikal" OR "RTXM-83" OR "RTXM83" OR "RTXM 83" OR "IDEC-102" OR "IDEC102" OR "IDEC 102" OR "IDEC-C2B8" OR "IDECC2B8" OR "IDEC C2B8" OR "IDEC-C2B8-anti-CD20" OR "MabThera" OR "RG 105" OR "RG-105" OR "RG105" OR "Ristova" OR "Rituxan" OR "RO-452294" OR "RO 452294" OR "RO452294" OR "GB-241" OR "GB 241" OR "GB241" OR "Blitzima" OR "CT-P10" OR "CT P10" OR "CTP10" OR "Ritemvia" OR "Rituzena" OR "Truxima" OR "Tuxella" OR "REDDITUX" OR "Reditux" OR "Tidecron") OR

("Rosiglitazone" OR "Avandia" OR "BRL 49653" OR "BRL 49653C") OR

("Rovelizumab" OR "23F2G" OR "Hu23F2G" OR "LeukArrest") OR

("tabalumab" OR "anti-BAFF Mab" OR "anti-BAFF monoclonal antibody" OR "anti-BAFF" OR "LY 2127399" OR "LY-2127399" OR "LY2127399") OR

("temelimab" OR "GENHP-01" OR "GNbAC1" OR "GNN001") OR

("tiplimotide" OR "CGP 77116" OR "CGP77116" OR "CGP-77116" OR "MSP 771" OR "MSP771" OR "MSP-771" OR "NBI 5788" OR "NBI5788" OR "NBI-5788") OR

("Toralizumab" OR "Anti-5c8 - IDEC" OR "Anti-CD154 - IDEC" OR "Anti-CD40L - IDEC" OR "Anti-gp39 - IDEC" OR "Anti-gp39 monoclonal antibody - IDEC" OR "Anti-T-BAM - IDEC" OR "Anti-TRAP - IDEC" OR "E 6040" OR "E-6040" OR "E6040" OR "IDEC 131" OR "IDEC131" OR "IDEC-131") OR

("CNTO 1275" OR "CNTO1275" OR "CNTO-1275" OR "ustekinumab" OR "anti-interleukin-12 p40 monoclonal antibody") OR

("vatelizumab" OR "CHR-1103" OR "CHR1103" OR "CHR 1103" OR "GBR 500" OR "GBR-500" OR "GBR500" OR "SAR 339658" OR "SAR-339658" OR "SAR339658" OR "TMC-2003" OR "TMC2003" OR "TMC 2003" OR "UNII-A4R7G50030" OR "UNIIA4R7G50030" OR "UNII A4R7G50030") OR

("cholecalciferol" OR "vitamin D3" OR "Vitamin-D3" OR "colecalciferol" OR "7-dehydrocholesterol" OR "Deltius" OR "Dibase" OR "Thorens" OR "Vitamin D") OR

("alemtuzumab" OR "BX 1523" OR "BX1523" OR "BX-1523" OR "BXT-1523" OR "BXT 1523" OR "BXT1523" OR "anti-CD52 monoclonal antibody" OR "Campath" OR "Campath-1H" OR "LDP-03" OR "LDP03" OR "LDP 03" OR "Lemtrada" OR "MabCampath" OR "MabKampat" OR "Remniq") OR

("cladribine" OR "2-chloro-2-deoxyadenosine" OR "2-chlorodeoxyadenosine" OR "chlorodeoxyadenosine" OR "Intocel" OR "Leustat" OR "Leustatin" OR "Mavenclad" OR "Movectro" OR "Mylinax" OR "PRMavenclad" OR "RWJ-26251" OR "RWJ26251" OR "RWJ 26251") OR

("dimethyl fumarate" OR "BG00012" OR "BG-00012" OR "BG 00012" OR "LAS-41008" OR "LAS41008" OR "LAS 41008" OR "Skilarence" OR "FP-187" OR "FP187" OR "FP 187" OR "BG-12 oral fumarate" OR "FAG-201" OR "FAG 201" OR "FAG201" OR "Panaclar" OR "Tecfidera") OR

("4-aminopyridine" OR "4-pyridinamine" OR "4-pyridylamine" OR "fampridine" OR "dalfampridine" OR "Fampyra" OR "BIIB041" OR "BIIB-041" OR "BIIB 041") OR

("fingolimod" OR "FTY-720" OR "FTY720" OR "FTY 720" OR "Gilenia" OR "Gilenya" OR "Imusera" OR "TDI-132" OR "TDI132" OR "TDI 132") OR

("glatiramer acetate" OR "Copolymer 1" OR "Cop-1" OR "Copaxone" OR "Timexon" OR "BCD-063" OR "BCD063" OR "BCD 063" OR "copolymer-1" OR "LBS-102" OR "LBS 102" OR "LBS102" OR "MyeloXen") OR

("interferon-beta" OR "Interferon beta-1a" OR "interferon beta 1-alpha" OR "Avonex" OR "Rebif" OR "Extavia" OR "betaseron" OR "BAY86-5046" OR "NU100" OR "NU-100" OR "NU 100" OR "Baronferon" OR "Relonsiv") OR

("ALKS 8700" OR "ALKS8700" OR "BIIB098" OR "monomethyl fumarate" OR "diroximel fumarate" OR "Bafiertam" OR "Vumerity" OR "AKLS-MMF" OR "ALKS MMF" OR "RDC-1559" OR "RDC1559" OR "RDC 1559") OR

("natalizumab" OR "Tysabri" OR "Antegren" OR "AN100226M" OR "N 10022" OR "N10022" OR "N-10022" OR "AN 100226" OR "AN-100226" OR "AN100226" OR "anti-alpha4 integrin monoclonal antibody" OR "BG-00002" OR "BG00002" OR "BG 00002" OR "BG-0002" OR "BG 0002" OR "BG0002" OR "BG0002-E" OR "BG 0002-E" OR "BG-0002-E" OR "DST-356A1" OR "DST356A1" OR "DST 356A1" OR "PB-006" OR "PB 006" OR "PB006") OR

("ocrelizumab" OR "Ocrevus" OR "2H7-monoclonal-antibody" OR "humanised anti-CD20 mAb" OR "monoclonal-antibody-2H7" OR "PRO 70769" OR "PRO-70769" OR "PRO70769" OR "R 1594" OR "R-1594" OR "R1594" OR "RG 1594" OR "RG-1594" OR "RG-1594" OR "rhuMab 2H7" OR "RO4964913" OR "RO-4964913" OR "RO 4964913") OR

("ofatumumab" OR "Arzerra" OR "Kesimpta" OR "GSK-1841157" OR "GSK1841157" OR "GSK 1841157" OR "HuMax CD20" OR "OMB-157" OR "OMB157" OR "OMB 157") OR

("ozanimod" OR "BMS-986374" OR "BMS986374" OR "BMS 986374" OR "RPC-1063" OR "RPC1063" OR "RPC 1063" OR "Zeposia") OR

("peginterferon beta-1a" OR "BIIB017" OR "BIIB-017" OR "BIIB 017" OR "Plegridy" OR "pegylated interferon beta-1a" OR "PEG-IFN-beta" OR "PEG-interferon-beta" OR "Peginterferon-beta" OR "PEGylated-interferon beta" OR "TRK-560" OR "TRK 560" OR "TRK560" OR "BCD-054" OR "BCD 054" OR "BCD054" OR "Tenexia") OR

("ponesimod" OR "ACT-128800" OR "ACT128800" OR "ACT 128800" OR "Compound 8bo" OR "Ponvory" OR "RG 3477" OR "RG3477" OR "RG-3477") OR

("siponimod" OR "BAF-312" OR "BAF312" OR "BAF 312" OR "Mayzent" OR "NVP BAF312 AEA" OR "NVP-BAF312-NX") OR

("teriflunomide" OR "Aubagio" OR "A-77-1726" OR "AVE-1726" OR "HMR-1726" OR "HMR1726D" OR "RS-61980" OR "SU-0020"))

AND

(Lysolecithin OR lysophosphatidylcholine OR "Ethidium bromide" OR "EtBr" OR Cuprizone OR "bis-cyclohexanone-oxaldihydrazone" OR "Anti-galactocerebroside antibodies" OR "Anti-galactocerebroside-antibodies" OR "anti-galactocerebroside" OR "Theiler׳s murine encephalomyelitis virus" OR TMEV OR "Experimental autoimmune encephalomyelitis" OR "EAE" OR "Experimental allergic encephalomyelitis"))

Databases searched: Medline via PubMed and Embase

Search interval: database inception until June 08, 2024

Number of retrieved records

-Medline: 2127

-Embase: 5177

After deduplication using the default Endnote tool: 6312 (see supplementary Figure 1 for details)

**References**

1. Hooijmans CR, Rovers MM, de Vries RB, Leenaars M, Ritskes-Hoitinga M, Langendam MW. SYRCLE's risk of bias tool for animal studies. *BMC medical research methodology* 2014; **14**: 43.

2. Zurrer WE, Cannon AE, Ewing E, Rosso M, Ineichen BV. Auto-STEED: A data mining tool for automated extraction of experimental parameters and risk of bias items from in vivo publications. *bioRxiv* 2023: 2023.02.24.529867.
